# Identification of Genes Differentially Expressed in Response to Cold in *Pisum sativum* Using RNA Sequencing Analyses

**DOI:** 10.3390/plants8080288

**Published:** 2019-08-15

**Authors:** Nasser Bahrman, Emilie Hascoët, Odile Jaminon, Frédéric Dépta, Jean-François Hû, Olivier Bouchez, Isabelle Lejeune-Hénaut, Bruno Delbreil, Sylvain Legrand

**Affiliations:** 1 ICV—Institut Charles Viollette, EA 7394, USC 1411, Univ. Lille, F-59000 Lille, France; 2 ICV—Adaptation au Froid du Pois, USC 1411, INRA, F-80300 Péronne, France; 3 GCIE, UE 0972, INRA, F-80203 Péronne, France; 4 GeT-PlaGe, Genotoul, US 1426, INRA, F-31326 Castanet-Tolosan, France; 5 Evo-Eco-Paleo, UMR 8198, Univ. Lille, CNRS, F-59000 Lille, France

**Keywords:** pea, cold stress, chilling, acclimation, freezing tolerance, transcriptome, RNA-seq

## Abstract

Low temperature stress affects growth and development in pea (*Pisum sativum* L.) and decreases yield. In this study, RNA sequencing time series analyses performed on lines, Champagne frost-tolerant and Térèse frost-sensitive, during a low temperature treatment versus a control condition, led us to identify 4981 differentially expressed genes. Thanks to our experimental design and statistical analyses, we were able to classify these genes into three sets. The first one was composed of 2487 genes that could be related to the constitutive differences between the two lines and were not regulated during cold treatment. The second gathered 1403 genes that could be related to the chilling response. The third set contained 1091 genes, including genes that could be related to freezing tolerance. The identification of differentially expressed genes related to cold, oxidative stress, and dehydration responses, including some transcription factors and kinases, confirmed the soundness of our analyses. In addition, we identified about one hundred genes, whose expression has not yet been linked to cold stress. Overall, our findings showed that both lines have different characteristics for their cold response (chilling response and/or freezing tolerance), as more than 90% of differentially expressed genes were specific to each of them.

## 1. Introduction

Cold stress is one of the most important factors that limit plant productivity around the world. Understanding the molecular bases of the cold response is thus essential to breed cold-tolerant varieties. To survive winter frosts, plants need to acquire frost tolerance which depends on the duration and time of the exposition to low temperatures [1] and varies according to species [2,3] and genotypes [4]. Plants can adopt two strategies to overcome frost. The first one consists of escaping the main frost periods, which can be obtained by different adaptive mechanisms in the natural population like developing a rosette form or reducing the hypocotyl length, which has been well documented in *Arabidopsis thaliana* [5,6,7]. The second one concerns the acquisition of freezing tolerance (FT), through a phenomenon called cold acclimation [8]. Following exposure to low temperatures, plants increase their ability to tolerate cold temperatures. Cold acclimation reveals two mechanisms of tolerance, which include: chilling tolerance and the induction of freezing tolerance (FT). Chilling tolerance represents the ability of a plant to respond to low but positive temperatures inferior to 15 ^◦^C, and FT is an induced response where plants acquire an increased freezing tolerance upon a prior low but non-freezing temperature treatment [9]. FT is a multigenic and quantitative trait that depends on more numerous metabolic changes than chilling [10]. The acquisition of freezing tolerance during acclimation depends on the duration and time of the exposition to cold [1], and varies according to species [11]. The cold acclimation process consists of a large number of changes at the molecular and metabolic levels [12,13].

Many transcriptomic studies have been undertaken in *A. thaliana* in order to decipher cold responses [14,15]. Some high throughput transcriptomic analyses have also been performed on cold stress in other plants, among which some have been conducted in legumes (*Fabaceae*) e.g., *Ammopiptanthus mongolicus* [16], *Glycine max* [17], *Lotus japonicas* [18], *Vignia unguiculata*, subspecies *sesquipedalis* [19], *Vigna subterranea* [20], *Medicago truncatula* [4], *Medicago falcata* [21], *Medicago sativa* [22,23], and *Cicer arietinum* [24]. All of these studies show, as in *A. thaliana*, the important role of transcription factors, including CBFs (C-repeat Binding Factors), kinases, and COR (Cold-Responsive) genes in cold regulation and acclimation. CBFs recognize and bind to cis-elements in the promoters of cold-COR genes, thus triggering their expression [25]. 

Among legumes, we are especially interested in dry pea (*Pisum sativum* L. (Ps)), which is an important source of proteins and starch for animal feeding and human food. Although dry peas are usually sown in spring in Europe, autumn sowings (winter peas) are desirable as they would allow for increasing and stabilizing the grain yield [26]. However, to permit autumn sowings, plants have to resist winter frost. In order to decipher the cold response in pea, we have already carried out transcriptomic approaches using microarray and suppression subtractive hybridization (SSH) [27,28]. However, these first approaches were limited by their relatively low throughput with only 11,930 non-cold specific ESTs available on the microarray and 5000 ESTs displayed within the SSH libraries. 

In this present investigation, using a RNA-sequencing (RNA-seq) approach, we took advantage of the Illumina high throughput technology, allowing us to detect low modulation of expression and to obtain a broader overview of the cold responses in pea. RNA-seq analyses performed on 24 mRNA libraries obtained from cold-treated and control samples of the pea lines Champagne (Ch) and Térèse (Te) led us to obtain nearly 900 million reads, and the resulting quantitative analysis allowed us to identify 793,583,651 clean reads ending up with 4981 differentially expressed genes (DEGs). The use of a known frost-tolerant line (Ch) and a frost-sensitive one (Te) allowed us, by comparing both lines submitted or not to a low temperature treatment, to classify DEGs into three sets: the first one corresponds to genes related to the constitutive differences between the two lines and that are not regulated during cold treatment (Line Response), the second one corresponds to genes in which expression is affected by the cold treatment and show similar expression patterns in the two lines (Temperature Common Response i.e., chilling response), and the last one corresponds to genes in which expression is affected by the cold treatment and present different patterns of expression in the two lines, including genes putatively related to FT (Temperature Line-Specific Response). 

## 2. Materials and Methods 

### 2.1. Plant Materials, Experimental Design, and Cold Stress

**Figure 1:**
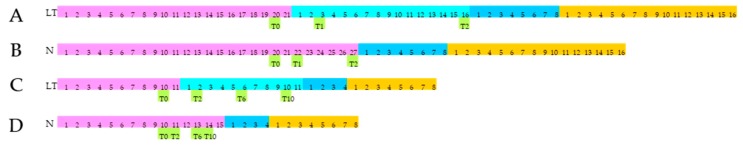
Scheme of the experiments and samplings. RNA-sequencing (RNA-seq) experiments (**A**): low temperature (LT) treatment and (**B**): control (N), nursery in magenta, low temperature in cyan, freezing in sky blue, and recovery period in orange; quantitative polymerase chain reaction (qPCR) experiments from Reference [28], as mentioned in § *2.5*, (**C**): LT and (**D**): N, color codes as in RNA-seq. The sampling dates are in green and the numbers refer to the days.

Two pea lines with contrasted characteristics for cold tolerance were subjected to a low temperature (LT) treatment. Ch is a frost-tolerant winter forage variety whereas Te is a frost-sensitive spring dry pea variety. Only Ch is able to cold acclimate and survive frost, while Te dies when submitted to negative temperatures even if prior subjected to a cold acclimation period [29]. Both lines were grown in a climatic chamber within eight isolating trays to prevent roots from freezing, each one having one hundred holes containing a Jiffy block. Both lines were grown in two different experiments respectively characterized by a LT period (Figure 1A) and by a control (N) temperature condition (Figure 1B). For the LT experiment, plants were first exposed to 20 °C day/14 °C night at 500 µmol m^−2^s^−1^ photosynthetic photon flux (PPF) with a 10 h photoperiod over 21 days. This initial phase was called the nursery period. It was followed by a LT period of 16 days with 8 °C day/2 °C night at 250 µmol m^−2^s^−1^ PPF and with a 10 h photoperiod. For the N experiment, the nursery period was extended up to 27 days, in order to be able to sample at the same developmental stages as in the LT experiment. In both experiments (LT and N), after the previous periods, the plants were exposed to freezing over 8 days at 4 °C day/−9 °C night at 150 µmol m^−2^s^−1^ PPF with a 10 h photoperiod. Afterwards, a recovery period of 16 days was applied with 16 °C day/5 °C night at 500 µmol m^−2^s^−1^ PPF with a 10 h photoperiod.

Three samplings were performed in each experiment (LT and N). For the N experiment, the samplings were performed at the 20th (T0), 22nd (T1), and 27th (T2) day of the nursery period. For the LT experiment the samplings were realized at the 20th day (T0) of the nursery period and at the 3rd (T1) and 16th (T2) day of the LT period (Figure 1). The developmental stage of the plants was regularly checked by the state of leaf unfolding, allowing the sampling of plants at the same developmental stage in both LT and N conditions, with the same final number of degree-days [30] despite differences in number of days until sampling. At the end of the recovery period, we confirmed the phenotype of the two lines, because the Ch plants survived the frost period, unlike the Te ones.

A total of 24 samples were harvested, corresponding to 2 lines × 2 treatments (LT and N) × 3 sampling times (T0, T1, and T2) × 2 biological replicates. For each sample, aerial parts of 3 plants were pooled and were immediately frozen using liquid nitrogen and stored at −80 °C until RNA extraction. 

### 2.2. RNA Extraction and High Throughput Sequencing

Total RNA was isolated with the Plant RNAeasy Mini kit (Qiagen) and quantified using a NanoDrop. The paired-end sequencing was performed using an Illumina HiSeq2000 sequencer at the Next Generation Sequencing (NGS) platform of GeT: http://get.genotoul.fr/. About 900 million raw reads generated from the 24 samples were deposited to the National Center for Biotechnology Information (NCBI) in the SRA (Short Read Archive) database (BioProject #PRJNA543764).

### 2.3. RNA-Sequencing Analysis, Assembly, and Annotation

The quality of the raw reads was checked using FastQC v0.11.4 [31]. After removing adapter sequences with Cutadapt v1.0 [32], reads were cleaned using Prinseq v0.20.3 [33]. The clean reads of the twenty-four samples were de novo assembled using Trinity v20140717 [34]. The contigs obtained by Trinity were filtered using TransRate v1.03 [35], which detects chimeric sequences, structural errors, incomplete assembly, and base errors. Then, Quast v2.3 [36] was used to assess the quality of the assembly. The transcriptome was annotated with Blastx searches [37] against *A. thaliana* (TAIR 10) protein databases.

Gene ontology (GO) (http://geneontology.org/ Gene Ontology Consortium and http://pantherdb.org/), and Kyoto Encyclopedia of Genes and Genomes (KEGG) (http://www.kegg.jp/kegg/tool/map_pathway2.html KEGG Mapper) information was assigned based on the *A. thaliana* homologous proteins, and GO functional classification was performed (https://www.arabidopsis.org/tools/bulk/go/index.jsp). Given the importance of protein kinases [38,39,40,41,42], transcription factors and transcription regulators (TF) [43,44,45,46] in cold stress responses, a specific annotation was made for these classes of genes by combining analyses from iTAK v1.2 (http://bioinfo.bti.cornell.edu/cgi-bin/itak/index.cgi) [47], PlnTFDB (http://plntfdb.bio.uni-potsdam.de/v3.0/), [48] and AGRIS (Arabidopsis Gene Regulatory Information Server) (http://arabidopsis.med.ohio-state.edu/, [49]).

### 2.4. Differential Expression Analysis and Statistical Tools

For the differential expression analysis, Illumina-cleaned reads from the 24 samples were pseudo-aligned on the de novo transcriptome assembly using Kallisto v0.43.1 [50]. Count data were analyzed using a multifactorial design (line (Ch and Te), treatment (LT and N), and time (T0, T1, and T2)) with the R package DESeq2 [51]. The multiple steps of statistical and clustering analyses led to three sets of distinct DEGs (Differentially Expressed Genes) (Figure S1). Firstly, only unigenes fulfilling the criterion TRUE (when the number of reads is sufficient to perform the statistical test) in DESeq2 were conserved for the next analyses. Then, unigenes represented by less than or equal to 48 normalized counts among the 24 samples were discarded. The *p*-values were calculated for nine combinations (ChNT0:TeNT0, ChNT0:ChNT1, ChNT0:ChNT2, ChLT0:ChLT1, ChLT0:ChLT2, TeNT0:TeNT1, TeNT0:TeNT2, TeLT0:TeLT1, and TeLT0:TeLT2). Only unigenes with an adjusted *p*-value ≤ 0.02 in at least one of the nine combinations were conserved. A sorting was then realized according to the significance of the functional annotation (*E*-value ≤ 9 × 10^−4^). The resulting set of unigenes was submitted to three successive analyses of variance (ANOVA) using Multi Experiment Viewer [52] statistical tools, in order to identify unigenes related to the differences between the two lines or related to the cold responses. First, a two factor ANOVA was performed allowing us to retain only DGEs which varied according to the line and/or the treatment. Then a one-way ANOVA was realized, providing a set of unigenes related to the differences between the two lines (Line Response). The remaining genes were submitted to an additional one-way analysis allowing us to decipher genes responding identically to the LT in the two lines (Temperature Common Response: TCR) from those which presented different cold responses in Ch and Te (Temperature Line Specific Response: TLSR).

Afterwards, gene expression patterns were built and classified with the MeV clustering tools. After log2 transformation of normalized count and mean-centered reduced fit, hierarchical clustering (HCL) were performed using Pearson’s correlation and average linkage clustering method. GO term enrichment analyses of the different sets of DEG were performed using AmiGO2 (http:/amigo1.geneontology.org/cgi-bin/amigo/term_enrichment).

### 2.5. Statistical Differentially Expressed Genes (DEGs) Corroboration

In order to support the expression of DEGs identified in this study, we used quantitative polymerase chain reaction (qPCR) data from an earlier published experiment [28] which presented only a few differences in the environmental conditions and the sampling times. The T0, T1, and T2 samples of the RNA-seq study were compared to the T0, T6, and T10 of the qPCR analysis, respectively (Figure 1). Blastn searches (*E*-value ≤ 1 × 10^−25^, coverage ≥ 200 pb, ≥ 95% of identity) were performed to link the unigenes representing DEGs in the RNA-seq study to the ESTs used to define primers for the qPCR analysis.

## 3. Results

### 3.1. Samples, Sequencing, and Assembly Assessment

Twenty-four RNA libraries were built from RNA samples extracted from the two contrasted pea lines, Ch (frost-tolerant) and Te (frost-sensitive), under LT (a low but non-freezing temperature regime is applied before submitting plants to frost) and N (frost is applied without any prior LT treatment). The Illumina sequencing of the 24 samples led us to obtain 886,477,626 paired-end reads. After removing low-quality sequences, a total of 793,583,651 clean reads were assembled into 150,342 contigs with a minimum length of 201 bp (Table S1). Following an analysis of the quality of the transcriptome assembly, 122,194 high-quality contigs, for a total of 118,787,279 bp and representing 73,225 unigenes were conserved for the further analyses. The largest contig was 11,608 bp long and the N50 value of the assembly was 1904 (Table S1). Blastx searches against *A. thaliana* protein sequences allowed to annotate 34% (24,854/73,225) of the unigenes (*E*-value ≤ 9 × 10^−4^). The distribution of the biological processes of the annotated unigenes resembled those reported for *A. thaliana* genes, suggesting that the construction of the pea RNA-seq libraries did not induce an enrichment of sequences related to a particular class of function (Figure S2).

### 3.2. Differential Expression Analysis and Clustering

**Figure 2:**
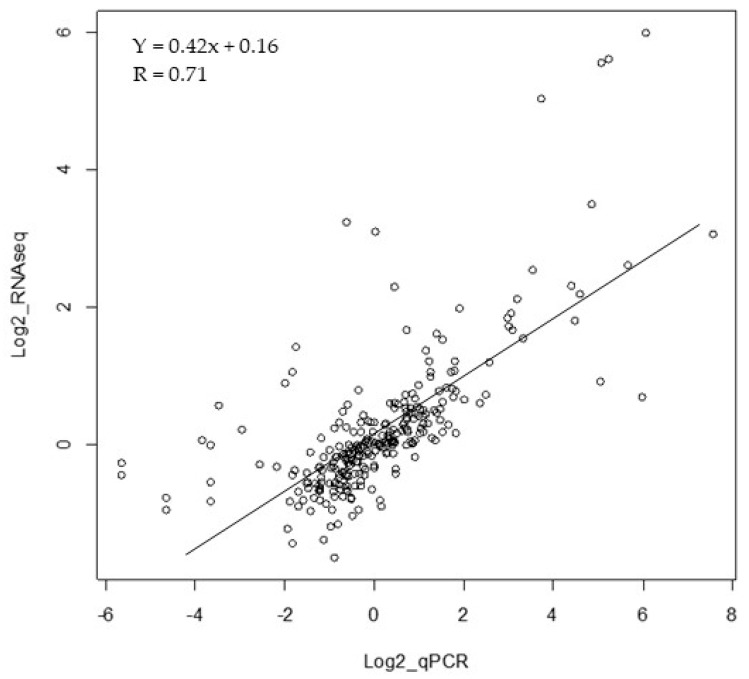
Scatter plot between the two sets of transcripts issued from the present RNA-seq and qPCR data from Reference [28]. The x-axis corresponds to log2 of qPCR ratios and the y-axis shows log2 of RNA-seq ratios for 33 transcripts. The linear relationship between the 2 variables and their correlation coefficient, R = ∑xy/√ ∑x^2^ ∑y^2^ are reported in the top of the graph.

The threshold applied on DESeq2 results (adjusted *p*-value ≤ 0.02) retained 11,076 unigenes, presenting a modulation of expression within the 24 different samples. Among them, 9676 unigenes were annotated using *A. thaliana* proteins. A two-way ANOVA performed on the 9676 annotated unigenes permitted us to detect 4981 genes, in which expression was significantly modulated according to the line and/or to the low temperature treatment (Figure S1). Among them, 33 were compared with data previously obtained by qPCR. We observed that RNA-seq and qPCR data are correlated (R = 0.71) (Figure 2). 

**Figure 3:**
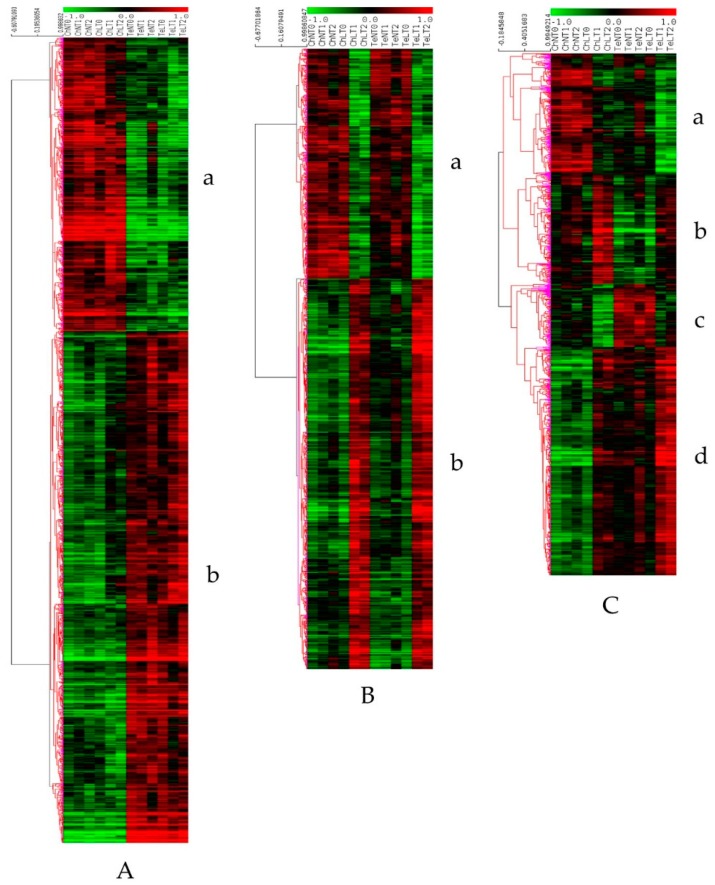
Hierarchical clustering of the three sets of differentially expressed genes. (**A**): “Line Response” containing two subsets, **a** (Ch up) and **b** (Te up); (**B**): “Temperature Common Response” having two subsets, a (TCR down) and b (TCR up) and (C): “Temperature Line Specific Response” including four subsets, TLSR a, b, c, and d. The order of the columns from left to right are ChNT0, ChNT1, ChNT2, ChLT0, ChLT1, ChLT2, TeNT0, TeNT1, TeNT2, TeLT0, TeLT1, and TeLT2.

The examination of the 33 expression patterns reflects the correlation between the data from the two methods of gene expression analysis and supports the robustness of the whole transcriptomic analysis (Figure S3). Then, the 4981 DEGs were submitted to a one-way ANOVA based on the line factor, which led to identify 2487 genes differentially expressed between Ch and Te but not modulated during the cold treatment (Line Response, LR). This set represents a part of the constitutive differences between the two lines. The hierarchical clustering (HCL) analysis divided this set into two blocks. The first block contained 906 genes, which were more expressed in the Ch than in the Te samples (LR (a), Figure 3A (a)) and inversely, the second one was composed of 1581 genes which were more expressed in the Te than in the Ch samples (LR b, Figure 3A (b)). Then a second one-way ANOVA based on the cold treatment factor was performed with the remaining 2494 (4981 − 2487 = 2494) DEGs (Figure S1) and revealed 1403 genes related to the “Temperature Common Response” (TCR) of the two lines. The HCL analysis carried out on these 1403 significant genes could separate 520 (Figure 3B (a)) and 883 (Figure 3B (b)) genes up-expressed in the N condition and the LT condition, respectively. The remaining 1091 genes (2494 − 1403 = 1091), which responded differently in Ch and Te under the LT treatment were considered to be associated to the “Temperature Line Specific Response” (TLSR). The HCL performed on this last set (Figure 3C) showed four distinct expression patterns. The first subset (TLSR (a)) gathered 253 genes which were more expressed in Ch than in Te and down-regulated during the LT conditions in both lines (Figure 3C (a)). The second subset (TLSR (b)) was composed of 228 genes which were also more expressed in Ch than in Te but up-regulated during the LT conditions (Figure 3C (b)). The third one (TLSR (c)) contained 131 genes that were less expressed in Ch than in Te and down-regulated during the LT conditions (Figure 3C (c)) and the last of the four subsets (TLSR (d)) was composed of 479 genes which were likewise less expressed in Ch than in Te but up-regulated during the LT treatment (Figure 3C (d)).

### 3.3. Functional Annotation, Gene Ontology (GO) Term Enrichment, and Kyoto Encyclopedia of Genes and Genomes (KEGG) Pathways

**Figure 4:**
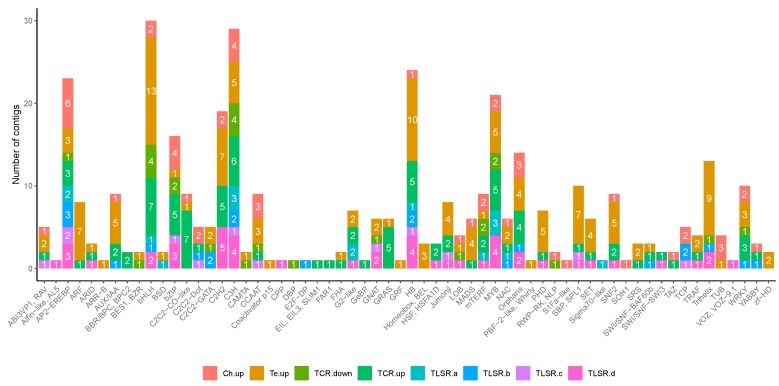
Transcription factors and regulators (TFs) compiled from iTAK data and AGRIS (Arabidopsis Gene Regulatory Information Server) (AtTFDB) in each subset of DEGs.

For the three sets of DEGs described above (LR, TCR, and TLSR) and distributed into eight subsets, the GO term enrichment analysis is detailed in Figure S4, the mapping on KEGG pathways in Table S2, the list of genes annotated as coding putative kinases in Table S3, and those annotated as coding transcription factors in Figure 4. In addition, a summary of all these results is provided in Table 1 and the list of genes known to be related to cold stress in Table 2. This comprehensive analysis of the function of DEGs allowed us to present an overview of the differences between the two lines and to decipher their respective cold responses.

We observed that the five most significant enriched GO terms related to biological processes were the same for all of the eight subsets. These terms were “metabolic process”, “cellular process”, “localization”, “cellular component organization or biogenesis”, and “response to stimulus”. This result seems to show that there is no difference in biological processes involved in the different subsets, at least not at this level of functional assignment. On the other hand, the enriched GO terms related to cellular localization were different within the subsets. In two cases, LR (a) and TCR (a), the five most significant terms related to localization concerned chloroplast and plastid components. This was also the case for a part of the terms enriched in the TLSR (a) subset. For all other subsets, the major enriched terms were related to cell, cytoplasm, and/or intercellular part.

The mapping of all DEGs on the KEGG pathways revealed relatively important differences in the number of mapped genes according to the subset (Table S2, Table 1). Indeed, 36% of the DEGs of the TLSR (d) subset were mapped on the pathways versus 80% of those of the TLSR (a) subset. This analysis, unlike the enrichment of GO terms, revealed a diversity of pathway representations between the subsets. For example, the most represented pathways in the LR (b) and TCR (b) subsets were related to the ribosome metabolism, while those of the LR (a) and TLSR (b) subsets were related to the amino acid metabolism.

The analysis, using iTak, allowed us to annotate 193 putative kinases among the DEGs of the three sets, including 96, 54, and 43 in the LR, TCR, and TLSR sets, respectively (Table S3, Table 1). Kinases were the most represented in the TLSR (b) and (d) subsets (4.8% for each) and the less represented in the TLSR (c) subset (1.5%). A total of 397 putative TFs was also annotated, with 188, 114, and 95 in the LR, TCR, and TLSR subsets, respectively (Figure 4, Table 1). TFs were the most represented in the TLSR (d) subset (9.6%) and the less represented in the TCR (a) subset (4.8%). The most represented family differs according to the subset, bHLH and AP2-EREBP being the most represented in three subsets each (LR (b), TCR (a) and (b) for bHLH and LR (a), TLSR (b) and (c) for AP2-EREBP). We noted that some of the kinases and TFs are known to be related to cold stress, including Myb, CBF, and WRKY (Table 2 and see below).

The functional classification using GO knowledgebase allowed us to classify 145 genes in GO terms related to the cold response, with 128, 11, and 6 in “response to cold” GO:0009409, “cold acclimation” GO:0009631, and “cellular response to cold” GO:0070417, respectively (Table 1, Table 2). Even if it was expected to identify genes related to these GO terms within the TCR (44 genes: 3.1%) and TLSR (36 genes: 3.3%) sets, it was more surprising to identify 65 genes (2.6%) in the LR set. This could mean that several genes that are known to be induced during cold stress in other species could be constitutively expressed in Ch or Te. The proportion of genes related to these GO terms in the different subsets was between 2.3% (LR (b)) and 6.9% (TLSR (c)).

## 4. Discussion

The genetic studies previously carried out by our team have shown that the Champagne and Térèse lines differ in their response to cold. Following a period of acclimation, the Ch line becomes tolerant to frost while Te remains sensitive [29,126]. This present study aims to provide molecular elements to explain the cold responses in pea. The statistical treatments of the RNA-seq data of 73,225 unigenes led us to distinguish 4981 DEGs, divided into three main sets according to their expression patterns. The analysis of these genes, their modulation of expression, and their affiliation to metabolic pathways has allowed us to enrich our knowledge on the behavior of these two lines regarding their response to cold. 

### 4.1. Differences in Gene Expression Between The Two Lines Independently of the LT Treatment: Predispositions to Face Cold Stress?

The class of genes considered here corresponds to genes which are differentially expressed in the two lines at T0, i.e., genes which are more expressed in Ch than in Te samples, or inversely. The opposition of gene expression patterns was observed throughout the time course study with an almost constant expression level in each line, independently of the LT treatment (Figure 3A). We can therefore consider these genes as being part of the constitutive differences between the two lines. In both Ch and Te up-expressed subsets (Figure 3A (a) and (b), respectively), the KEGG pathway annotation revealed that a majority of these genes are related to metabolism (84.4% and 68.9% for the Ch and Te up-expressed subsets, respectively) suggesting important intrinsic differences between the two lines regarding cellular metabolism. In particular, 73 and 49 genes were related to amino-acid and energy metabolisms among genes up-expressed in Ch and Te, respectively. In addition, 102 genes in the Ch up-expressed subset and 39 in the Te up-expressed subset were related to RNA and protein metabolism. The GO-enrichment analyses were consistent with these results, highlighting the assignment of genes related to metabolism and to RNA and RNA surveillance pathways in the Ch and Te up-expressed subsets, respectively. Interestingly, even if the majority of the genes considered here did not present any modulation of expression during the time course study, 50/906 (5.5%) and 108/1581 (6.8%) of genes from the Ch and Te up-expressed subsets respectively, are known to be involved in responses to diverse stimuli, notably to responses to stress. In particular, within the Ch up-expressed subset, we identified genes coding a cold-regulated 413 inner membrane protein 1 (AT1G29395), and a 3-oxoacyl-synthase II (AT1G74960), which are known to accumulate in cold/freezing conditions in order to preserve chloroplast membranes integrity [110,111]. We also noted in the Ch up-expressed subset the presence of two genes, the first being a mediator of the RNA polymerase II transcriptional subunit 32 (AT1G11760) which regulates the expression of the second, a dehydration element B1A (CBF3, AT4G25480). Both are well known for their involvement in cold acclimation in *A. thaliana* [112]. More surprisingly, several genes are also known to be involved in the cold responses within the Te up-subset. For example, we found in this subset three genes coding AGAMOUS-like proteins (AT3G61120, AT4G22950, AT2G45660), known to lead to early flowering in *A. thaliana* and induced by an extended cold treatment [127]. We also identified genes coding a calmodulin-binding transcription activator protein (AT5G64220), which is involved in the rapid induction of CBF factors [53], and an adenine nucleotide alpha hydrolase-like super family (AT1G09740), which enhances freezing tolerance in *Arabidopsis* after a short period of cold-acclimation [54]. Moreover, genes coding a raffinose synthase (AT5G20250, [45]), a xyloglucan endotransglucosylas/hydrolase (AT4G03210 [55]), and an enolase (in pea [128] and LOS2, AT2G36530 [56]) were scored in the Te up-expressed subset, all of them being involved in cold stress in *A. thaliana*. The fact that some genes related to cold stress are up-expressed in Te could also be expected, since this line could possess deficient alleles that would not confer to Te an effective defense against cold.

Concerning the kinases, we observed within the Ch up-expressed subset one gene encoding a MPK3 (mitogen-activated protein kinase 3, AT3G45640) shown to be up-expressed in response to cold stress in *A. thaliana* [57]. Within the Te up-expressed subset, we identified another MAP3K (MAPK/ERK kinase kinase 1, AT4G08500) that plays a major role in cold stress signaling in *A. thaliana* [113]. More interestingly, 50 (5.52%) and 138 (8.73%) TFs were identified in Ch and Te up-expressed subsets, respectively. Among them, 50 distinct TF families were represented, including 19 TF families which gathered preferentially expressed genes in Ch and Te (i.e., common TF families) and 7 and 24 families which were represented by genes preferentially expressed in Ch or Te, respectively (i.e., line-specific TF families). Among them, several TFs were previously signaled as differentially expressed in cold stress in legume species [16,18,19,22] or are known to be involved in cold acclimation or freezing tolerance in *A. thaliana,* e.g., DREB1 (CBF3), CAMTA, NAC, and WRKY [25,129]. The other TFs, including ARID, BSD, mTERF, RWP-RK, S1Fa-like, SOH1, SRS, SWI/SNF-BAF60b, TAZ, and TUB are reported for the first time in pea and may be also involved in the intrinsic phenotypic differences between the two lines. 

A recent study, using genetic structure and linkage disequilibrium in a large collection of pea germplasm, also highlighted the line-dependent differences [130]. These differences may be due to the fact that Ch belongs to the winter forage lines cohort and Te is issued from the spring lines set. Overall, our analysis reflects the importance of intrinsic differences between lines at the gene expression level [28,128,131]. Among this “Line Response” set, many genes coding proteins related to cold responses were identified. These results suggest that Ch and Te have constitutive defenses against cold stress, which have been described in the majority of cases as induced in other species and particularly in *A. thaliana*. 

### 4.2. How Pea Faces Cold Stress

The identification of genes showing a significant increase/decrease of expression in Ch and Te during the LT and no fluctuation in the N experiment suggests that both lines have the capacity to undertake molecular modifications in response to cold stress, that can be considered as the chilling response shared by both genotypes. These genes were gathered into the TCR set. In addition, genes showing variation of expression only in Ch during the LT treatment could be attributed to the FT capacity of this frost tolerant line and were clustered within the TLSR set. Elsewhere, the expression pattern variations are more diverse for this set of TLSR, both at the beginning of the LT and also over time, as well as in N condition between these two lines. Furthermore, it is important to note that for both TCR and TLSR sets, we have scored more up-expressed genes in Te than in Ch.

#### 4.2.1. Chilling Response

The TCR set was separated into two subsets, gathering almost the same number of genes which were down- or up-regulated during the LT treatment. Concerning down-expressed genes, it should be noted that the enrichment of GO terms related to chloroplast could provide evidence that genes involved in the photosynthetic system are hugely affected by chilling (Figure S4 (F)). These observations are in agreement with the fact that cold stress leads to a disruption and/or dysfunction of photosynthesis and causes damages to thylakoid membrane and chloroplastic envelopes [132]. The up-expressed subset, for its part gathered, in particular, several RNA-binding proteins, which are involved in RNA and RNA surveillance pathways, operating in plant responses to abiotic stress [58,133]. Concerning the GO annotations, we observed that 30 (3.40%) and 14 (2.7%) genes of the up- and down-expressed subsets respectively, were associated to cold (Table 2). For example, genes representing a STARCH EXCESS 1 (AT1G10760 [114]), a Glycine-rich RNA-binding protein7 (AT2G21660 [59]), two low temperature and salt-responsive protein LTI6A and LTI6B (AT3G05880 and AT3G05890 [60]), a Late Embryogenesis Abundant 4-5 (AT5G06760 [61]), and a Galactinol synthase 2 (AT1G56600 of the raffinose pathway [62]) were found in the up-expressed subset and are known to present an increase of expression in response to cold condition in *A. thaliana*. At last, a transcriptional adapter ADA2b (AT4G16420), which may repress freezing tolerance and does not require the expression of CBF or COR genes in *A. thaliana* [115] was scored in the up-subset. Moreover, we found also in the up-expressed subset genes coding a VERNALIZATION INDEPENDENCE 4 protein (AT5G61150, homologous to LEO1), involved in vernalization response in many *A. thaliana* ecotypes [134] and a sensitive to freezing 2 protein (AT3G06510, SFR2, a constitutively expressed b-glucosidase), which is conserved in all land plants [63] and involved in the response to freezing by protecting chloroplast membrane from damages. 

Overall, concerning kinases and TFs annotations, we observed that most of the annotated kinases in up- and down-expressed subsets are known to be differentially expressed in cold stress [16,18,19,22]. In particular, we identified a gene coding calcium/calmodulin-regulated receptor-like kinase 1 (AT5G54590) in the up-expressed subset that is involved in freezing tolerance in *A. thaliana* [113], and 5 casein kinases I and 3 SnRK which were involved in stress response [38,135]. Elsewhere, more TFs were scored in the up- than in the down-expressed subset, with seven families being represented in both subsets, and eight and 31 families being specific to down- or up-expressed subsets, respectively. In particular, we identified specifically into the up-expressed subset a MADS protein (AT4G24540, AGAMOUS-LIKE 24 protein), known to be implicated as a transcription activator mediating floral transition in response to vernalization [7]. Here, we report for the first time the involvement of genes coding ARID, DBP, EIL3, mTERF, RWP-RK, SWI, and TAZ proteins in the chilling response. 

#### 4.2.2. Champagne Specific Responses to Cold and Acquisition of Freezing Tolerance

As we have seen previously, the specific response to cold concerns a greater variety of genes than the common response. We observed, whatever the modulation of gene expression levels (increase or decrease), many genes presented different level of expression in Ch and Te at the starting point of the time course study (T0). These observations again highlight the importance of the constitutive differences, even in the specific responses of the two lines. Indeed, the second subset (TLSR (b), Figure 3C (b)) gathered up-regulated genes in Ch and in Te during LT, but which are more expressed in Ch, as a result of a higher expression level at T0 for this line. Similarly, genes within the third subset (TLSR (c), Figure 3C (c)) are down-regulated during LT and are less expressed in Ch than in Te during LT as a result of a lower level of expression in Ch at T0. Hence, genes from clusters TLSR (b) and TLSR (c) could be related to the specific responses of Ch taking part in the freezing tolerance capacity of this line. In a same manner, first and forth subsets (Figure 3C (a) and (d)) gathered genes that are related to the specific responses of Te. Considering that these responses (qualitatively and/or quantitatively) are ineffective to bring freezing tolerance to Te, they won’t be discussed below. We noted that only very few genes presented a modulation of expression between the two sampling times (3 and 16 days) during LT, suggesting that at day three, most of the defenses against cold stress are implemented.

Within the TLSR (b) subset, six genes were assigned to GO terms related to cold including a gene coding a pre-mRNA-processing factor 31 homolog (AT1G60170, PRP31), which is involved in the regulation of expression of cold-responsive genes (*COR*s) in *A. thaliana* [120]. The PRP31 protein possesses a HAT domain, which is also found in 4 tetratricopeptide repeat protein (AT3G53560, AT2G37400, AT3G46790 and AT3G23020, TPR and/or PRP1) present in this subset [64]. The five other genes code a RNA-binding family protein (AT1G70200, presenting RRM/RBD/RNP motifs), which is implicated in cold tolerance by 23S ribosomal RNA processing in *A. thaliana* [64], two chloroplastic extern membrane protein 16-1 (AT2G28900) induced in low temperature in land plants [65], a Fibrillin-1a (AT4G04020) which is involved in response to freezing [66], and a Glycine-rich RNA-binding protein 2 (AT4G13850), which confers freezing tolerance after a cold acclimation period [116]. The KEGG annotation revealed that 28.3% of the genes were mapped on other pathways than those related to “Metabolism” and were rather related to RNA and RNA-related pathways. Thus, the RNA metabolism and more particularly RNA-binding with two Pentatricopeptide repeat-containing (AT1G11290 and AT1G20300, PPR motif), one RNA-binding (AT3G08620, KH domain) family, two RNA-binding (AT1G70200 and AT3G20890, PRM/RBD/RNP motifs), one Helicase (AT3G08620, KH domain), and one Glycine-rich containing domain (AT4G13850, RRM domain) proteins in this subset seem to be an important component of the specific cold response in Ch pea line, as well as in *A. thaliana* [133,136] and *Oryza sativa* [137]. 

The post-translational regulation using kinase proteins is considered as a key feature in plant response against cold stress [138]. Among the 11 up-expressed genes coding kinases in the subset TLSR b, two code calcium-dependent protein kinase 6 (AT4G14580 [57] and AT2G17290 [139]) and one a receptor-like protein kinase FERONIA (AT3G51550 [140]) that are involved in stomatal closure control in relation to cold conditions. Moreover, a gene coding a leucine-rich repeat receptor-like serine/threonine-protein kinase implicated in jasmonic acid and ethylene-dependent systemic resistance (AT3G14840 [141]) is scored. In the earlier studies [28,142], the evidence that jasmonate metabolism could play a role in freezing tolerance was provided. TF constitute 9.21% of the genes of this subset. Excepted SWI/SNF-BAF60b, which is signaled in this study for the first time in cold responses, all other are already known to be up or down regulated under cold treatment in legumes species [16,19,22].

The KEGG annotation within the TLSR (c) subset revealed that most of the genes were associated to “Metabolism”. A total of 9 genes were assigned to GO terms related to cold, including genes coding an acyl-CoA-binding domain-containing protein 6 (AT1G31812) that binds phosphatidylcholine in phospholipid metabolism [143], a chlorophyll a/b-binding protein 3-1, chloroplastic (AT1G61520) belonging to the light-harvesting complex in photosystem I [67], two NADPH-dependent aldo-keto reductase, chloroplastic (AT2G37770) that detoxifies a range of toxic aldehydes and ketones produced during stress [68], a bidirectional sugar transporter SWEET17 (AT4G15920) involved in fructose transport [121], and a 3-hydroxyisobutyryl-CoA hydrolase 1 (AT5G65940) that plays a role in peroxisomal metabolism in cold stress signaling and plant tolerance to cold stress, by the degradation of valine [69]. We noticed also the presence of two genes coding a PLAT domain-containing protein 1 (AT4G39730), which functions as positive regulator of abiotic stress tolerance [70], a serine/thereonine protein kinase 2 (AT3G08720), which is a downstream effector of the target of rapamycin signaling pathway (TOR) that presents an increase of protein activity via a phosphorylation induced under cold treatment [71]. All of the TF of TLSR (c) subset were already recorded in cold and freezing tolerance in legumes species, validating once again their participation as key factors of the cold acclimation process in Ch.

## 5. Conclusions 

In most published studies dealing with cold acclimation, gene expression analyses have been realized at the beginning (1 to 3 h) [12,14] or within the 24 h [19] after the acclimation period. Since we look at gene expression after three and sixteen days of acclimation, we expected to identify novel differentially expressed genes. Hence the expressed genes in this work should be involved in subsequent stages of the cold response and more downstream of metabolic chains.

From a total of 4489 differentially expressed genes, we observed the importance of the constitutive differences in gene expression between the two lines. In particular, we identified more preferentially expressed genes related to RNA metabolism in Te, and to protein metabolism in Ch.

Elsewhere, we observed that most of the genes we identified as involved in the freezing tolerance presented similar modulations (activation/repression) in Ch and Te, but with different levels of expression at the beginning of the time course study. This reveals again the importance of initial differences, in the specific responses of the two lines. According to the examination of clusters gathering genes related to chilling and FT, we were surprised to observe less genes modulated by the cold stress in Ch compared to Te. This probably means that Ch presents constitutive and/or induced mechanisms that are more efficient to get over cold than those implemented in Te.

Furthermore, we have identified many TFs, which are linked here for the first time to cold responses. Overall, genes whose expression is for the first time correlated with cold response could open new horizons in the use of genetic diversity of low temperature responses in pea. 

## Figures and Tables

**Table 1:** Summary of the functional annotation.

Set	Subset Description	# Genes	5 Most Significant Enriched GO Terms Related to Biological Process	5 Most Significant Enriched GO Terms Related to Cellular Localization	Kegg Pathways: # Mapped Genes (%), # Represented Pathways, Most Represented Pathways (#)	iTak: # Kinases (%), # Families	Transcription Factors: # TF (%), # Families, Most Represented Family (#)	GO Terms Related to Cold: # Genes
Line response (LR): genes differentially expressed between Ch and Te at T0 and not modulated under LT	Genes more expressed in Ch than in Te (Figure 3A a)	906	metabolic process, cellular process, localization, cellular component organization or biogenesis, response to stimulus	plastid, chloroplast cytoplasmic part, cell part, cell	553 (61.0%), 110, Cysteine and methionine metabolism (13)	26 (3.0%), 12	50 (5.5%), 26, AP2-EREBP (6)	-response to cold GO:0009409: 23 -cold acclimation GO:0009631: 4 -cellular response to cold GO:0070417: 2 -total: 29 (3.2%)
	Genes more expressed in Te than in Ch(Figure 3A b)	1581	cellular process, metabolic process, localization, cellular component organization or biogenesis, response to stimulus	cell part, cell, intracellular part, intracellular and intracellular organelle	616 (39.0%), 106, Ribosome (25)	70 (4.4%), 19	138 (8.7%) 43, bHLH (13)	-response to cold GO:0009409: 35 -cellular response to cold GO:0070417: 1 -total: 36 (2.3%)
Temperature Common Response (TCR): Genes responding identically in Ch and Te under LT	Genes down expressed during LT (Figure 3B a)	520	metabolic process, cellular process, cellular component organization or biogenesis, localization, response to stimulus	chloroplast, plastid, chloroplast part, plastid part, cytoplasm	366 (70.4%), 77, Glyoxylate and dicarboxylate metabolism (10)	16 (3.1%), 12	25 (4.8%), 16, bHLH (4)	-response to cold GO:0009409: 14 -Total: 14 (2.7%)
	Genes up expressed during LT (Figure 3B b)	883	metabolic process, cellular process, cellular component organization or biogenesis, localization, response to stimulus	intracellular, intracellular part, cell part, cell, membrane-bounded organelle	388 (43.9%), 88, Ribosome biogenesis (21)	38 (4.3%), 22	89 (10.1%), 38, bHLH (7), C2C2-CO-like (7)	-response to cold GO:0009409: 25 -cold acclimation GO:0009631: 3 -cellular response to cold GO:0070417: 2 -total: 30 (3.4%)
Temperature Line Specific Response (TLSR): genes responding differentially in Ch and Te under LT	Genes more expressed in Ch than in Te and down regulated during LT (Figure 3C a)	253	metabolic process, cellular process, localization, cellular component organization or biogenesis, response to stimulus	cytoplasmic part, plastid, chloroplast, cytoplasm, intracellular organelle part	201 (79.5%), 73, Oxidative phosphorylation (6), Starch and sucrose metabolism (6)	7 (2.8%), 6	16 (6.3%), 10, C3H (3), MYB (3)	-response to cold GO:0009409: 8 -cold acclimation GO:0009631: 1 -total: 9 (3.6%)
	Genes more expressed in Ch than in Te and up regulated during LT (Figure 3C b)	228	cellular process, metabolic process, localization, response to stimulus, cellular component organization or biogenesis	cell part, cell, intracellular part, cytoplasm, intracellular	170 (74.6%), 62, Purine metabolism (5)	11 (4.8%), 10	21 (9.2%), 15, AP2-EREBP (3)	-response to cold GO:0009409: 4 -cold acclimation GO:0009631: 1 -cellular response to cold GO:0070417: 1 -total: 6 (2.6%)
	Genes less expressed in Ch than in Te and down regulated during LT (Figure 3C c)	131	metabolic process, cellular process, localization, response to stimulus, cellular component organization or biogenesis	cytoplasmic part	57 (43.5%), 34, several pathways represented by 2 genes	2 (1.5%), 2	12 (9.2%), 11, AP2-EREBP (2)	-response to cold GO:0009409: 8 -cold acclimation GO:0009631: 1 -total: 9 (6.9%)
	Genes less expressed in Ch than in Te and up regulated during LT (Figure 3C d)	479	metabolic process, cellular process, localization, cellular component organization or biogenesis, response to stimulus	cell part, cell, intracellular part, intracellular, nucleus	173 (36.1%), 71, mRNA surveillance pathway (7)	23 (4.8%), 16	46 (9.6%), 25, C2H2 (5)	-response to cold GO:0009409: 11 -cold acclimation GO:0009631: 1 -total: 12 (2.5%)

Ch: Champagne; LT: low temperature treatment; Te: Térèse; TF: transcription factor.

**Table 2:** GO matching of the cold response (i.e., “response to cold”, “cold acclimation”, “cellular response to cold”) in eight subsets of differentially expressed genes. The Ch (Champagne) up and Te (Térèse) up columns correspond to “LR”, followed by two columns of “TCR” down and up and the last four columns referring to the “TLSR” a, b, c, and d, as described in the legend of Figure 3.

Protein/Gene/Function	Class of Function	Reference	Orthologous in AT	Ch Up	Te Up	TCR Down	TCR Up	TLSR a	TLSR b	TLSR c	TLSR d
*GO:0009409 (response to cold)*											
Calmodulin-binding transcription activator 2	TF, Induction of CBFs	[53]	AT5G64220.2		1						
Plasma-membrane cation-binding protein 1	Plasma membrane protein	[54]	AT4G20260.6		1						
Vacuolar H(+)-ATPase subunit E1	Hydrogen ion transport	[54]	AT4G11150.1				1				
Adenine nucleotide alpha hydrolases-like	Cold shock response	[54]	AT3G53990.1		2						
Calcium-dependent lipid-binding protein	Response to cold	[54]	AT4G34150.1		1						
SAUR-like auxin-responsive protein family	Auxin metabolism	[55]	AT4G38840.1		4	4					
Enolase, ENO2	Glycolysis/Gluconeogenesis	[56]	AT2G36530.1		1						
MAP kinase kinase kinase1	Kinase activity	[57]	AT4G08500.1		1						
Protein HAPLESS 6, Ribophorin II	N-linked glycosylation	[58]	AT4G21150.3		1						
Cold, circadian rhythm, RNA-binding 2, GRP7	RNA-binding	[59]	AT2G21660.2	5	5		5				
Glycine-rich RNA-binding protein 3, RBG3	RNA-binding, transcription	[59]	AT5G61030.1	2							
Hydrophobic protein RCI2A and LTI6A	Response to cold	[60]	AT3G05880.1				2				
Late embryogenesis abundant protein 46	Cryoprotectant	[61]	AT5G06760.1				1				
Galactinol synthase 2, GOLS2	Galactose metabolism	[62]	AT1G56600.1				1				
Protein sensitive to freezing 2	Glucosidase activity	[63]	AT3G06510.1				1				
Pentatricopeptide repeat-containing protein	RNA modification, binding	[64]	AT3G22690.2								1
RNA-binding (RRM/RBD/RNP motifs)	rRNA-binding	[64]	AT1G70200.1						1		
Outer envelope pore protein 16-1, OEP16-1	Amino acid transport, porin	[65]	AT2G28900.1	2					2		
Glutathione S-transferase F8	Oxidoreductase, peroxidase	[66]	AT2G47730.1			1					
Glyoxalase I	methylglyoxal degradation	[66]	AT1G67280.2		2						
Plastid-lipid-associated protein 1, Fibrillin-1a	Photoinhibition	[66]	AT4G04020.1						1		
Phosphoribulokinase, PRK	Photosynthesis, transferase	[66]	AT1G32060.1			1					
NADPH-dependent alkenal/one	Oxidoreductase	[66]	AT1G23740.1	2		2					
Serine hydroxymethyltransferase	One-carbon metabolism	[66]	AT4G37930.1			1					
RuBisCo activase	ATP- and nucleotide-binding	[66]	AT2G39730.1		1						
Chlorophyll a-b binding protein 4, LHCA4	Photosynthesis	[67]	AT3G47470.1		1					1	
NADPH-dependent aldo-keto reductase	Oxidation-reduction process	[68]	AT2G37770.2				2			2	
3-hydroxyisobutyryl-CoA hydrolase 1	L-valine degradation	[69]	AT5G65940.1							1	
PLAT domain-containing protein 1, PLAT1	Catalase, Peroxidase activity	[70]	AT4G39730.1	2						2	
Serine/threonine protein kinase	ATP binding, transferase	[71]	AT3G08720.2							1	
Phosphoglyceromutase 1, PGAM 1	Glycolysis/Gluconeogenesis	[72]	AT1G09780.1				1				
3-ketoacyl-CoA synthase 1	Fatty acid elongation	[73]	AT2G26250.1	1		2		2			
Acyl-CoA-binding protein 1, ACBP1	Fatty Acid Beta-Oxidation	[74]	AT5G53470.1				1			1	
Agamous-like MADS-box protein, SOC1	Transcription regulation	[75]	AT2G45660.1		2						
Alcohol dehydrogenase 1	Glycolysis/Gluconeogenesis	[76]	AT1G77120.1	1							
Annexin D8, calcium/phospholipid binding	Calcium binding	[77]	AT5G12380.1		1	1					1
BAG family molecular chaperone regulator 4	Chaperone binding	[78]	AT3G51780.1	1							
Calcium-binding protein	Calcium ion binding	[79]	AT1G02270.1	1							
Heat shock 70 KDa protein 1, HSP70-1	ATPase activity, chaperone	[79]	AT5G02500.1		2						
DNA damage-repair/toleration, DRT102	Isomerase activity	[79]	AT3G04880.1		1						
Proteasome subunit alpha type-3, PAG1	Folding, sorting, degradation	[79]	AT2G27020.1				1				
Hsp 70 kDa protein 1	ATPase activity, Chaperone	[79]	AT5G02500.1					2			
Calmodulin-binding receptor-like, kinase 1	Calmodulin binding	[80]	AT5G58940.1								1
Serine/threonine-protein kinase	ATP-binding	[81]	AT1G01140.3								1
Chaperonin-like RBCX protein 1	Protein folding chaperone	[82]	AT4G04330.1		1						
Cinnamoyl-CoA reductase 1	Lignin biosynthesis	[83]	AT1G15950.1					1			
Cold regulated protein 27, COR27	Cold, circadian rhythm	[84]	AT5G42900.3				1				
Cysteine proteinase inhibitor 6, CYS6	Cysteine proteinase inhibitor	[85]	AT3G12490.2				1				
Protein CRYOPHYTE, RH38	RNA-binding, hydrolase	[86]	AT3G53110.1								1
Diacylglycerol kinase 2	Glycerolipid metabolism	[87]	AT5G63770.1				1				
E3 ubiquitin-protein ligase HOS1	Protein ubiquitination	[88]	AT2G39810.1								1
Early light-induced protein 1, Chloroplastic	Photosynthesis	[89]	AT3G22840.1	1							
Ethylene-responsive TF, RAP2-4 and RAP2	Transcription factor	[90]	AT1G78080.1		1		1				
Glycine-rich RNA-binding protein RZ1A	RNA-binding, transcription	[91]	AT3G26420.1				1				1
HVA22-like protein a, similarity to TB2/DP1	Cold and stress response	[92]	AT1G74520.1	1			1				
Inositol-1-monophosphatase	Myo-inositol biosynthesis	[93]	AT3G02870.3	1							
Lipid transfer protein EARLI 1	Lipid-transfer	[94]	AT4G12480.1				1				
LOW-TEMPERATURE-INDUCED 65, LTI65	Response to abscisic acid	[95]	AT5G52300.2				1				
MAP kinase 3	ATP binding	[96]	AT3G45640.1	1							
MYB-related transcription factor CCA1	DNA binding	[97]	AT2G46830.1					2			
Phosphoinositide phospholipase C1	Hydrolase, lipid metabolism	[98]	AT5G58670.1		1						
Phospholipase D delta	Lipid degradation	[99]	AT4G35790.2		2						2
Protein EARLY FLOWERING 3, ELF3	DNA-binding TF activity	[100]	AT2G25930.1			1					
Protein ESKIMO 1, Signal-anchor	Xylan O-acetyltransferase	[101]	AT3G55990.1					1			
Protein GIGANTEA, GI	Phytochrome B signaling	[102]	AT1G22770.1								1
Protein Senescence-Associated Gene 21	Oxidative stresses	[103]	AT4G02380.1	2							
Raffinose synthase 6	Carbohydrate metabolism	[104]	AT5G20250.4		1						
Synaptotagmin-1, SYT1	Lipid binding, Ca^2+^ transport	[105]	AT2G20990.1		1						
Transcription factor GTE10, NPX1	ABA signaling pathway	[106]	AT5G63320.1								1
Tubulin beta-6 chain, TUBB6	GTPase activity, Transport	[107]	AT5G12250.1			1					
WRKY DNA-binding protein 33, WRKY33	DNA-binding TF activity	[108]	AT2G38470.1				1				
Xyloglucan endotransglucosylase/hydrolase protein 22	Glycosidase, Transferase	[109]	AT5G57560.1		1						
*GO:0009631 (cold acclimation)*											
Cold-regulated 413 thylakoid membrane 1	Cellular response to cold	[110]	AT1G29395.1	1							
3-oxoacyl-[acyl-carrier-protein] synthase II	Fatty acid biosynthesis	[111]	AT1G74960.3	1							
Mediator of RNA polymerase II transcription subunit 32	Transcription regulation	[112]	AT1G11760.1	1							
Calcium/calmodulin-regulated receptor-like kinase 1	Calmodulin-binding	[113]	AT5G54590.2				1				
Alpha-glucan water dikinase 1	Carbohydrate metabolism	[114]	AT1G10760.1				1				
Transcriptional adapter ADA2b, PRZ1	Transcription regulation	[115]	AT4G16420.1				1				
Glycine-rich RNA-binding protein 2	Chaperone, RNA-binding	[116]	AT4G13850.3						1		
Acetyl-CoA carboxylase 1	Fatty acid metabolism	[117]	AT1G36160.2					1			
C-repeat binding factor 3, DREB1A, CBF3	TF, DNA-binding	[118]	AT4G25480.1	1							
VOZ1, vascular plant one zinc finger	Transcription factor	[119]	AT1G28520.2								1
*GO:0070417 (cellular response to cold)*											
Pre-mRNA-processing factor 31 homolog	RNA-binding, splicing	[120]	AT1G60170.1						1		
Bidirectional sugar transporter SWEET17	Fructose, sugar transport	[121]	AT4G15920.1							1	
Delta(8)-fatty-acid desaturase1	Oxidoreductase	[122]	AT2G46210.1	1			1				
Glutamate receptor 3.4, GLR3.4	Ion transport	[123]	AT1G05200.2				1				
Organic cation/carnitine transporter 3, OCT3	Transporter activity	[124]	AT1G16390.1		1						
spliceosome protein-like protein	RNA processing, splicing	[125]	AT1G54380.1	1							
Total of genes in GO bulk analyses				29	36	14	30	9	6	9	12
Total of genes in each subset				906	1581	520	883	253	228	131	479
Ratio %				3.20	2.28	2.69	3.40	3.56	2.63	6.87	2.51

## Supplementary Materials

The following are available online at https://www.mdpi.com/2223-7747/8/8/288/s1, Figure S1: Workflow for statistical analyses, Figure S2: Functional classification by Gene Ontology, Figure S3: Comparison of the qPCR and the RNA-seq data, Figure S4: GO terms enrichment of Biological Processes and Cellular Component, Table S1: Statistical overview of sequencing and transcriptome assembly data, Table S2: KEGG pathways repartition and classification, Table S3: Kinases repartition in each subset of DEGs.
