# Supplementary material for: Identification of Genes Differentially Expressed in Response to Cold in Pisum sativum Using RNA Sequencing Analyses
Source: Plants (Basel). 2019 Aug 15;8(8):288. doi: 10.3390/plants8080288 (PMC6724123; doi:10.3390/plants8080288)
Supplement: Supplementary file 1 [file plants-08-00288-s001.pdf]

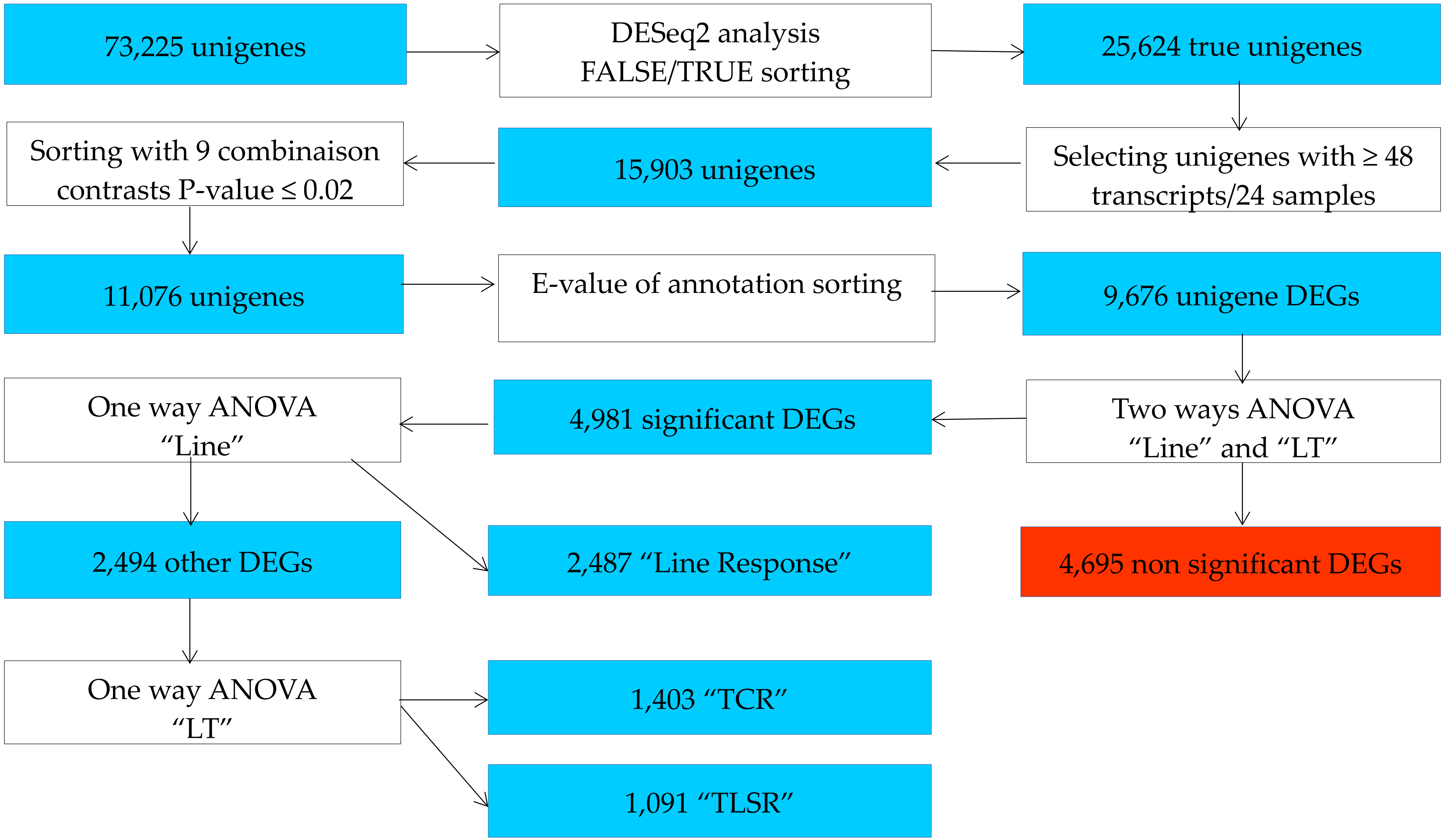

Figure S1. Statistical analysis and clustering workflow leading to three sets of differentially expressed genes (DEGs). Blue colored boxes show the gene numbers, uncolored boxes represent statistical analyses, and red box show non significant DEGs.

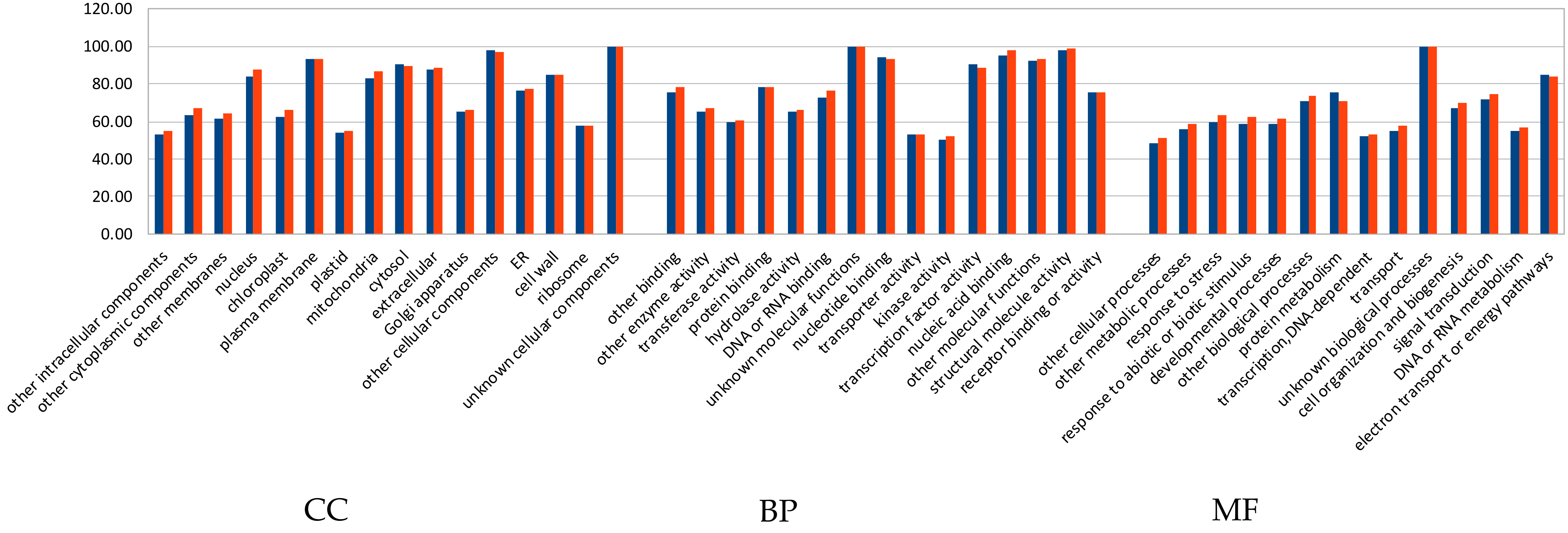

Figure S2. Functional classification of assembled unigenes carried out using Gene Ontology. The distribution of the cellular component **(CC)**, molecular function **(MF)** and biological processes **(BP)**, obtained from the whole transcriptome assembly (blue bars) is compared with that of the *A. thaliana* whole genome (red bars).

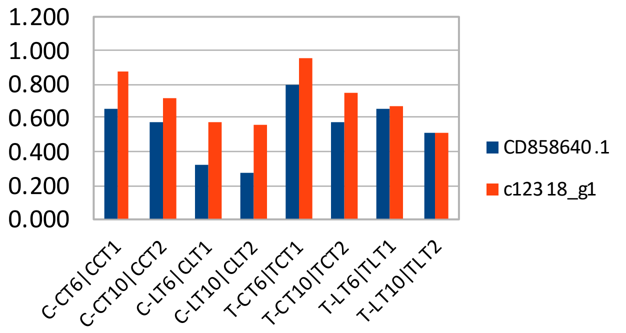
1.200
1.000
0.800
0.600
0.400
0.200
0.000
CD858640.1
c12318_g1
C-CT6|CCT1
C-CT10|CCT2
C-LT6|CLT1
C-LT10|CLT2
T-CT6|TCT1
T-CT10|TCT2
T-LT6|TLT1
T-LT10|TLT2

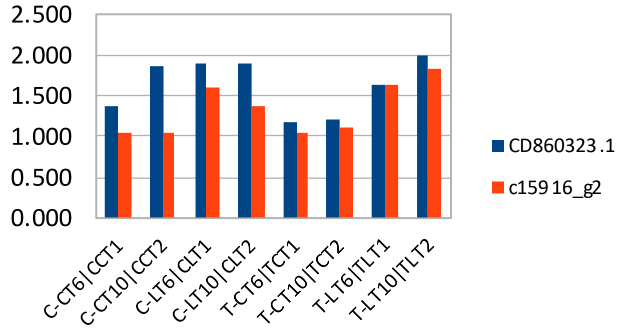
2.500
2.000
1.500
1.000
0.500
0.000
CD860323.1
c15916_g2
C-CT6|CCT1
C-CT10|CCT2
C-LT6|CLT1
C-LT10|CLT2
T-CT6|TCT1
T-CT10|TCT2
T-LT6|TLT1
T-LT10|TLT2

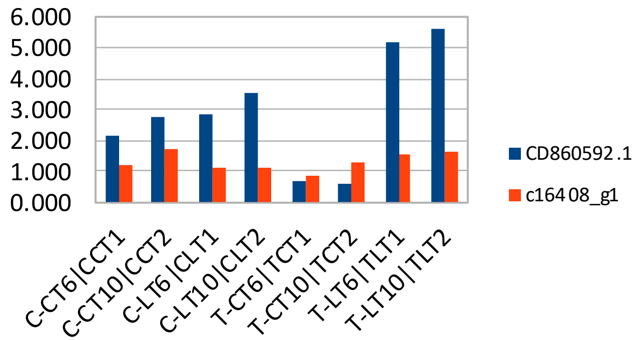
6.000
5.000
4.000
3.000
2.000
1.000
0.000
CD860592.1
c16408_g1
C-CT6|CCT1
C-CT10|CCT2
C-LT6|CLT1
C-LT10|CLT2
T-CT6|TCT1
T-CT10|TCT2
T-LT6|TLT1
T-LT10|TLT2

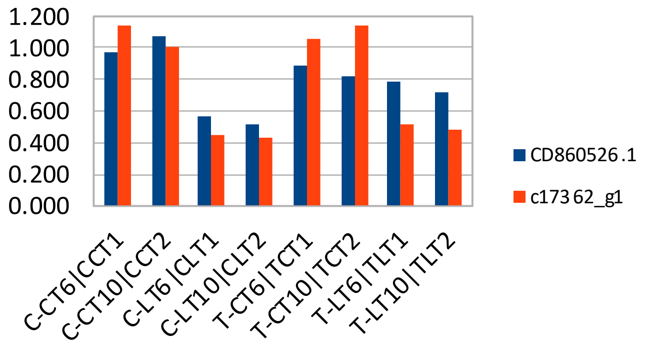
1.200
1.000
0.800
0.600
0.400
0.200
0.000
CD860526.1
c17362_g1
C-CT6|CCT1
C-CT10|CCT2
C-LT6|CLT1
C-LT10|CLT2
T-CT6|TCT1
T-CT10|TCT2
T-LT6|TLT1
T-LT10|TLT2

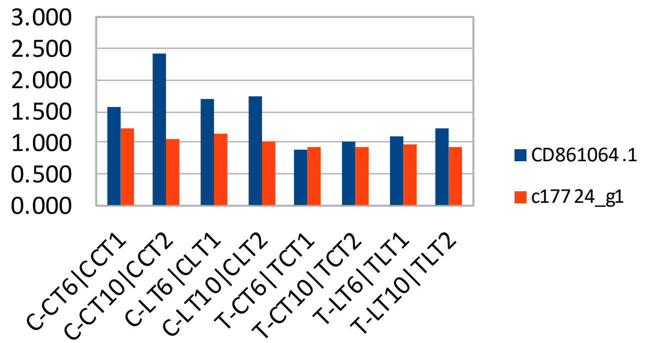
3.000
2.500
2.000
1.500
1.000
0.500
0.000
CD861064.1
c17724_g1
C-CT6|CCT1
C-CT10|CCT2
C-LT6|CLT1
C-LT10|CLT2
T-CT6|TCT1
T-CT10|TCT2
T-LT6|TLT1
T-LT10|TLT2

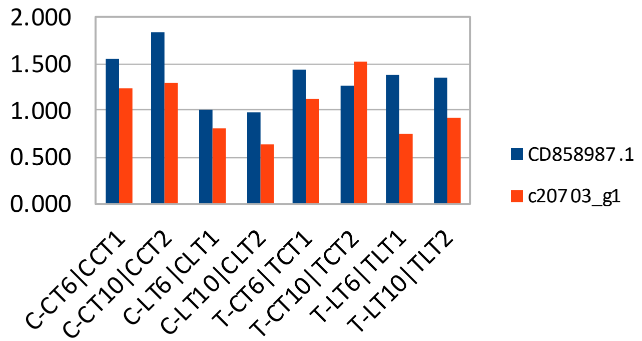
2.000
1.500
1.000
0.500
0.000
CD858987.1
c20703_g1
C-CT6|CCT1
C-CT10|CCT2
C-LT6|CLT1
C-LT10|CLT2
T-CT6|TCT1
T-CT10|TCT2
T-LT6|TLT1
T-LT10|TLT2

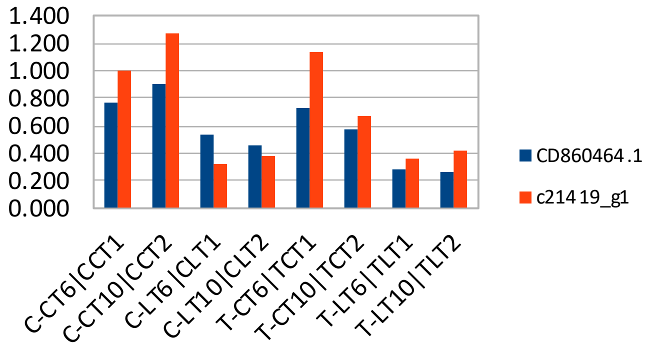
1.400
1.200
1.000
0.800
0.600
0.400
0.200
0.000
CD860464.1
c21419_g1
C-CT6|CCT1
C-CT10|CCT2
C-LT6|CLT1
C-LT10|CLT2
T-CT6|TCT1
T-CT10|TCT2
T-LT6|TLT1
T-LT10|TLT2

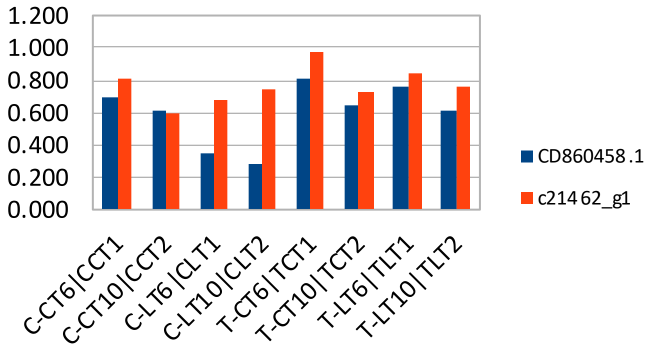
1.200
1.000
0.800
0.600
0.400
0.200
0.000
CD860458.1
c21462_g1
C-CT6|CCT1
C-CT10|CCT2
C-LT6|CLT1
C-LT10|CLT2
T-CT6|TCT1
T-CT10|TCT2
T-LT6|TLT1
T-LT10|TLT2

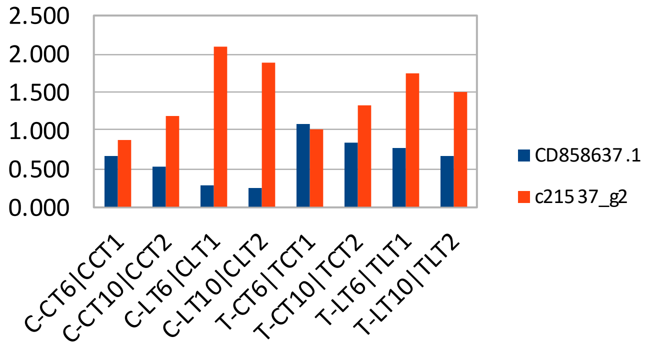
2.500
2.000
1.500
1.000
0.500
0.000
CD858637.1
c21537_g2
C-CT6|CCT1
C-CT10|CCT2
C-LT6|CLT1
C-LT10|CLT2
T-CT6|TCT1
T-CT10|TCT2
T-LT6|TLT1
T-LT10|TLT2

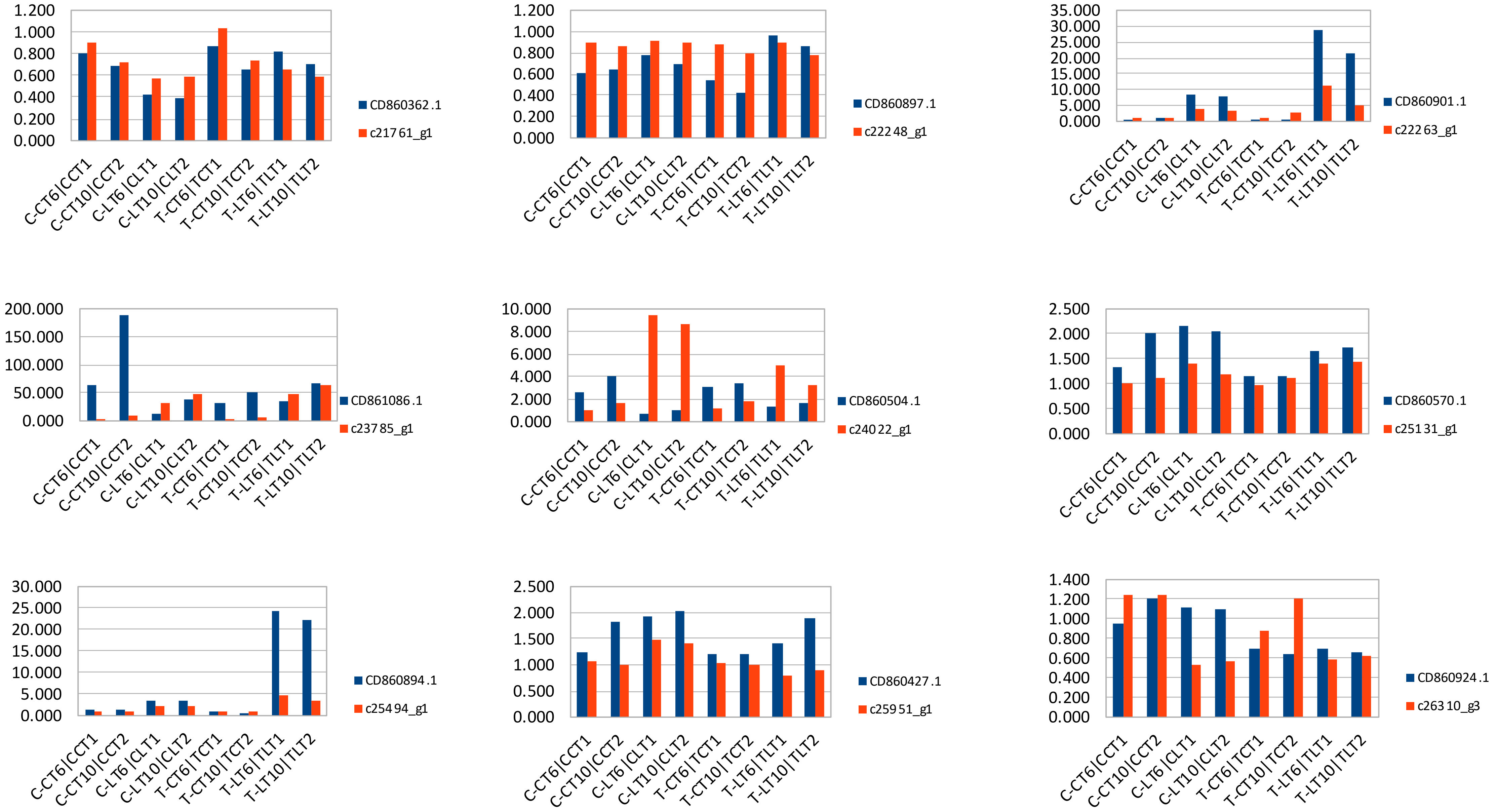
1.200
1.000
0.800
0.600
0.400
0.200
0.000
C-CT6|CCT1
C-CT10|CCT2
C-LT6|CLT1
C-LT10|CLT2
T-CT6|TCT1
T-CT10|TCT2
T-LT6|TLT1
T-LT10|TLT2
CD860362.1
c21761_g1
1.200
1.000
0.800
0.600
0.400
0.200
0.000
C-CT6|CCT1
C-CT10|CCT2
C-LT6|CLT1
C-LT10|CLT2
T-CT6|TCT1
T-CT10|TCT2
T-LT6|TLT1
T-LT10|TLT2
CD860897.1
c22248_g1
35.000
30.000
25.000
20.000
15.000
10.000
5.000
0.000
C-CT6|CCT1
C-CT10|CCT2
C-LT6|CLT1
C-LT10|CLT2
T-CT6|TCT1
T-CT10|TCT2
T-LT6|TLT1
T-LT10|TLT2
CD860901.1
c22263_g1
200.000
150.000
100.000
50.000
0.000
C-CT6|CCT1
C-CT10|CCT2
C-LT6|CLT1
C-LT10|CLT2
T-CT6|TCT1
T-CT10|TCT2
T-LT6|TLT1
T-LT10|TLT2
CD861086.1
c23785_g1
10.000
8.000
6.000
4.000
2.000
0.000
C-CT6|CCT1
C-CT10|CCT2
C-LT6|CLT1
C-LT10|CLT2
T-CT6|TCT1
T-CT10|TCT2
T-LT6|TLT1
T-LT10|TLT2
CD860504.1
c24022_g1
2.500
2.000
1.500
1.000
0.500
0.000
C-CT6|CCT1
C-CT10|CCT2
C-LT6|CLT1
C-LT10|CLT2
T-CT6|TCT1
T-CT10|TCT2
T-LT6|TLT1
T-LT10|TLT2
CD860570.1
c25131_g1
30.000
25.000
20.000
15.000
10.000
5.000
0.000
C-CT6|CCT1
C-CT10|CCT2
C-LT6|CLT1
C-LT10|CLT2
T-CT6|TCT1
T-CT10|TCT2
T-LT6|TLT1
T-LT10|TLT2
CD860894.1
c25494_g1
2.500
2.000
1.500
1.000
0.500
0.000
C-CT6|CCT1
C-CT10|CCT2
C-LT6|CLT1
C-LT10|CLT2
T-CT6|TCT1
T-CT10|TCT2
T-LT6|TLT1
T-LT10|TLT2
CD860427.1
c25951_g1
1.400
1.200
1.000
0.800
0.600
0.400
0.200
0.000
C-CT6|CCT1
C-CT10|CCT2
C-LT6|CLT1
C-LT10|CLT2
T-CT6|TCT1
T-CT10|TCT2
T-LT6|TLT1
T-LT10|TLT2
CD860924.1
c26310_g3

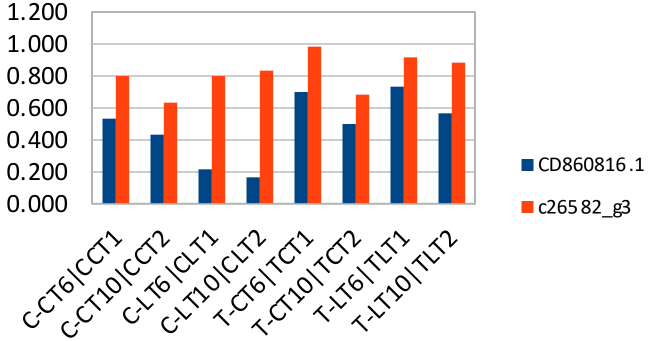
1.200
1.000
0.800
0.600
0.400
0.200
0.000
C-CT6|CCT1
C-CT10|CCT2
C-LT6|CLT1
C-LT10|CLT2
T-CT6|TCT1
T-CT10|TCT2
T-LT6|TLT1
T-LT10|TLT2
CD860816.1
c26582_g3

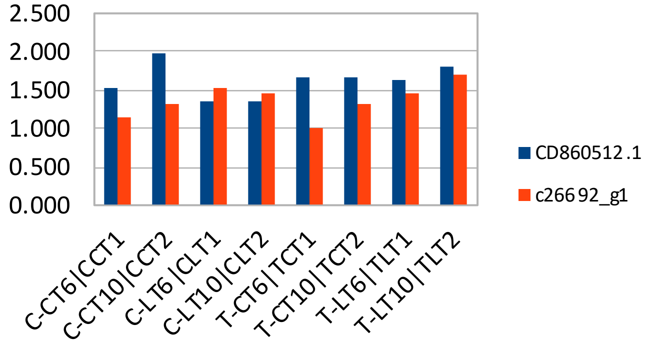
2.500
2.000
1.500
1.000
0.500
0.000
C-CT6|CCT1
C-CT10|CCT2
C-LT6|CLT1
C-LT10|CLT2
T-CT6|TCT1
T-CT10|TCT2
T-LT6|TLT1
T-LT10|TLT2
CD860512.1
c26692_g1

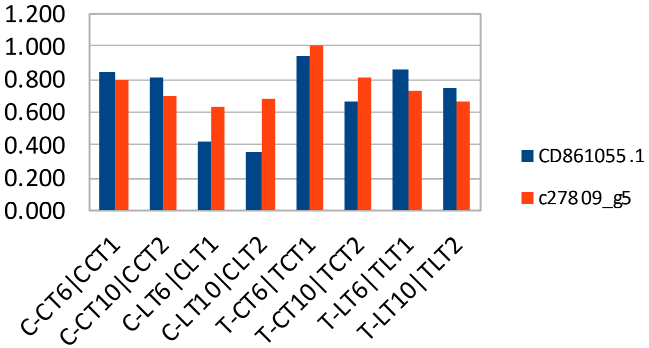
1.200
1.000
0.800
0.600
0.400
0.200
0.000
C-CT6|CCT1
C-CT10|CCT2
C-LT6|CLT1
C-LT10|CLT2
T-CT6|TCT1
T-CT10|TCT2
T-LT6|TLT1
T-LT10|TLT2
CD861055.1
c27809_g5

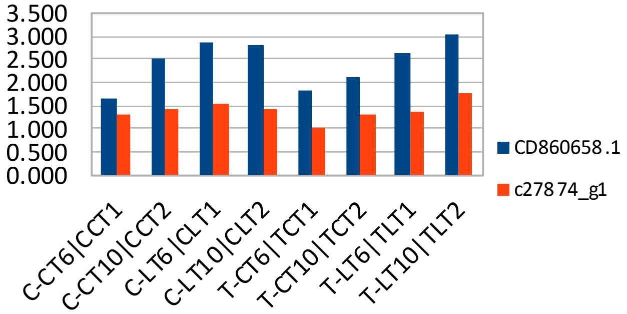
3.500
3.000
2.500
2.000
1.500
1.000
0.500
0.000
C-CT6|CCT1
C-CT10|CCT2
C-LT6|CLT1
C-LT10|CLT2
T-CT6|TCT1
T-CT10|TCT2
T-LT6|TLT1
T-LT10|TLT2
CD860658.1
c27874_g1

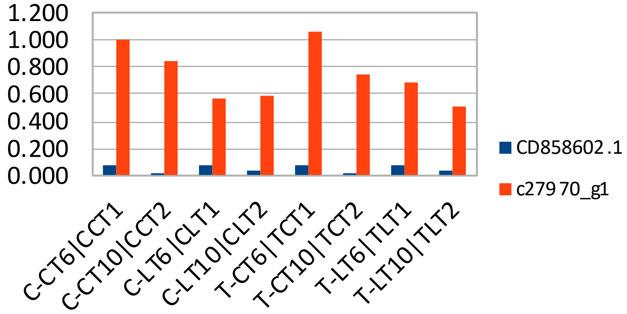
1.200
1.000
0.800
0.600
0.400
0.200
0.000
C-CT6|CCT1
C-CT10|CCT2
C-LT6|CLT1
C-LT10|CLT2
T-CT6|TCT1
T-CT10|TCT2
T-LT6|TLT1
T-LT10|TLT2
CD858602.1
c27970_g1

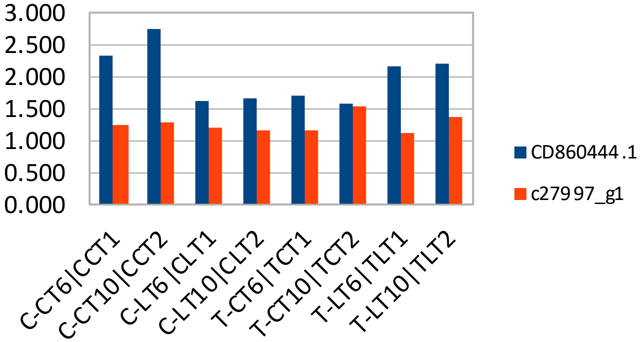
3.000
2.500
2.000
1.500
1.000
0.500
0.000
C-CT6|CCT1
C-CT10|CCT2
C-LT6|CLT1
C-LT10|CLT2
T-CT6|TCT1
T-CT10|TCT2
T-LT6|TLT1
T-LT10|TLT2
CD860444.1
c27997_g1

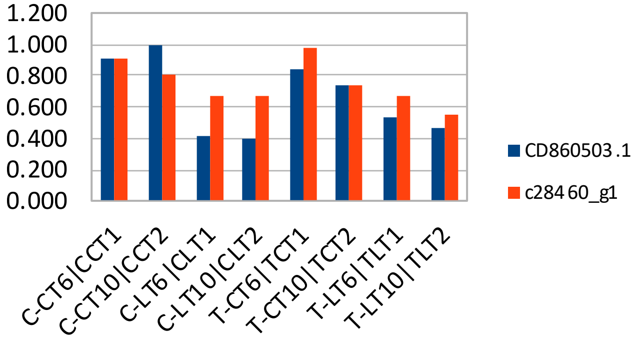
1.200
1.000
0.800
0.600
0.400
0.200
0.000
C-CT6|CCT1
C-CT10|CCT2
C-LT6|CLT1
C-LT10|CLT2
T-CT6|TCT1
T-CT10|TCT2
T-LT6|TLT1
T-LT10|TLT2
CD860503.1
c28460_g1

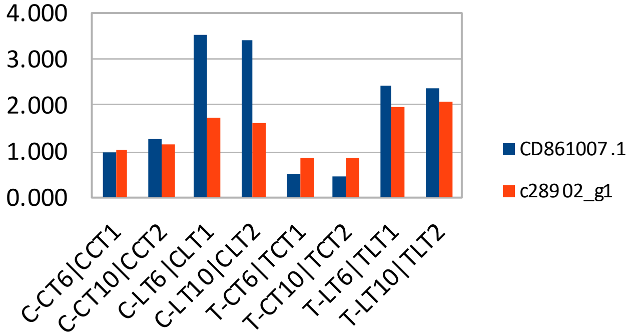
4.000
3.000
2.000
1.000
0.000
C-CT6|CCT1
C-CT10|CCT2
C-LT6|CLT1
C-LT10|CLT2
T-CT6|TCT1
T-CT10|TCT2
T-LT6|TLT1
T-LT10|TLT2
CD861007.1
c28902_g1

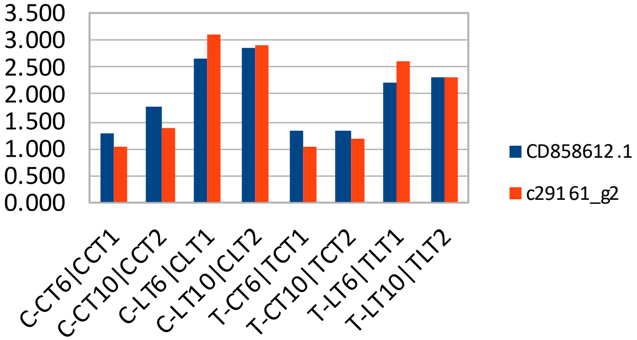
3.500
3.000
2.500
2.000
1.500
1.000
0.500
0.000
C-CT6|CCT1
C-CT10|CCT2
C-LT6|CLT1
C-LT10|CLT2
T-CT6|TCT1
T-CT10|TCT2
T-LT6|TLT1
T-LT10|TLT2
CD858612.1
c29161_g2

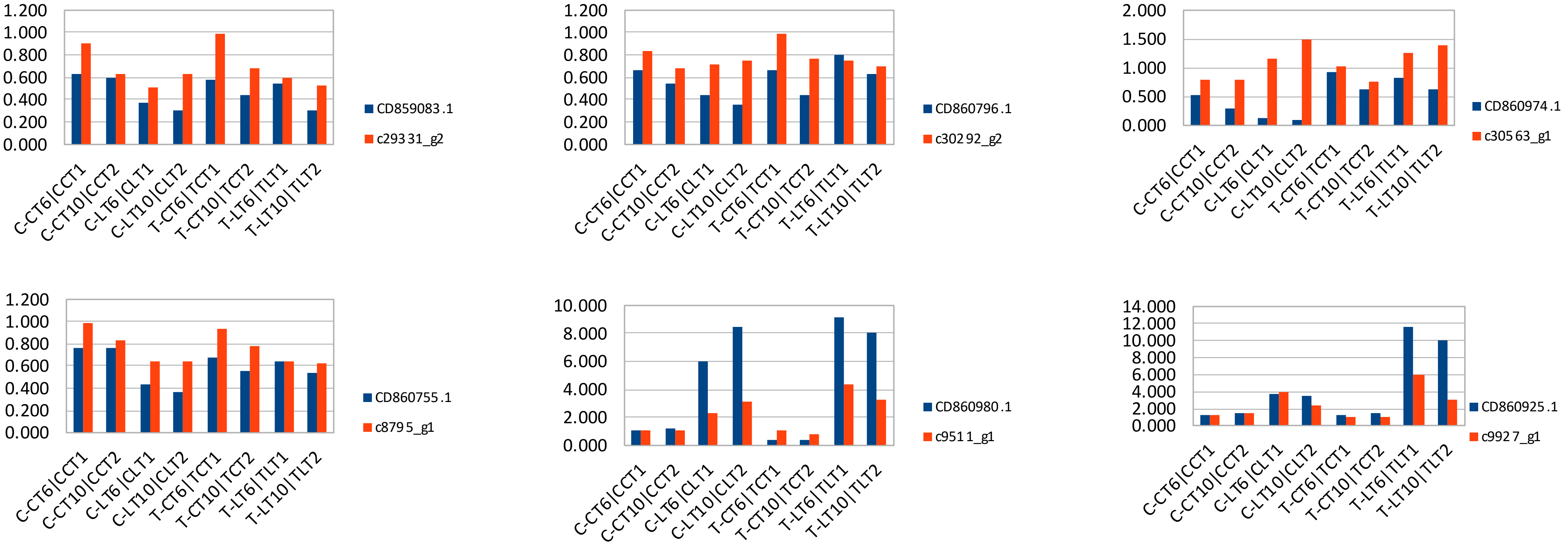

Figure S3. Individually comparisons of thirty-three qPCR and RNAseq data. Blue and red bars represent the qPCR and the RNA-seq data, respectively. For more details, see figure 1 and figure 2.

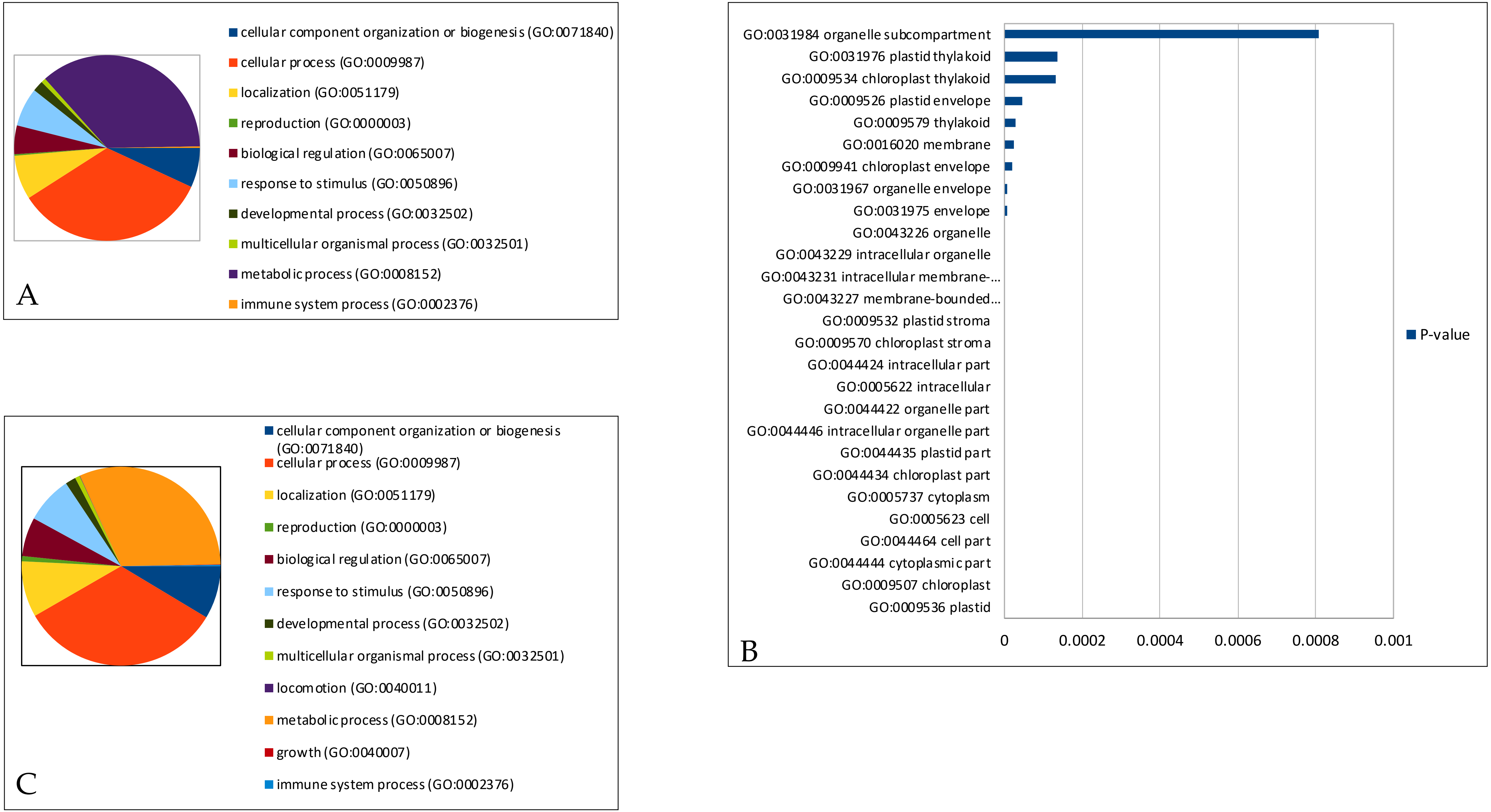

cellular component organization or biogenesis (GO:0071840)
cellular process (GO:0009987)
localization (GO:0051179)
reproduction (GO:0000003)
biological regulation (GO:0065007)
response to stimulus (GO:0050896)
developmental process (GO:0032502)
multicellular organismal process (GO:0032501)
metabolic process (GO:0008152)
immune system process (GO:0002376)
A
GO:0031984 organelle subcompartment
GO:0031976 plastid thylakoid
GO:0009534 chloroplast thylakoid
GO:0009526 plastid envelope
GO:0009579 thylakoid
GO:0016020 membrane
GO:0009941 chloroplast envelope
GO:0031967 organelle envelope
GO:0031975 envelope
GO:0043226 organelle
GO:0043229 intracellular organelle
GO:0043231 intracellular membrane-...
GO:0043227 membrane-bounded...
GO:0009532 plastid stroma
GO:0009570 chloroplast stroma
GO:0044424 intracellular part
GO:0005622 intracellular
GO:0044422 organelle part
GO:0044446 intracellular organelle part
GO:0044435 plastid part
GO:0044434 chloroplast part
GO:0005737 cytoplasm
GO:0005623 cell
GO:0044464 cell part
GO:0044444 cytoplasmic part
GO:0009507 chloroplast
GO:0009536 plastid
0
0.0002
0.0004
0.0006
0.0008
0.001
P-value
B
cellular component organization or biogenesis (GO:0071840)
cellular process (GO:0009987)
localization (GO:0051179)
reproduction (GO:0000003)
biological regulation (GO:0065007)
response to stimulus (GO:0050896)
developmental process (GO:0032502)
multicellular organismal process (GO:0032501)
locomotion (GO:0040011)
metabolic process (GO:0008152)
growth (GO:0040007)
immune system process (GO:0002376)
C

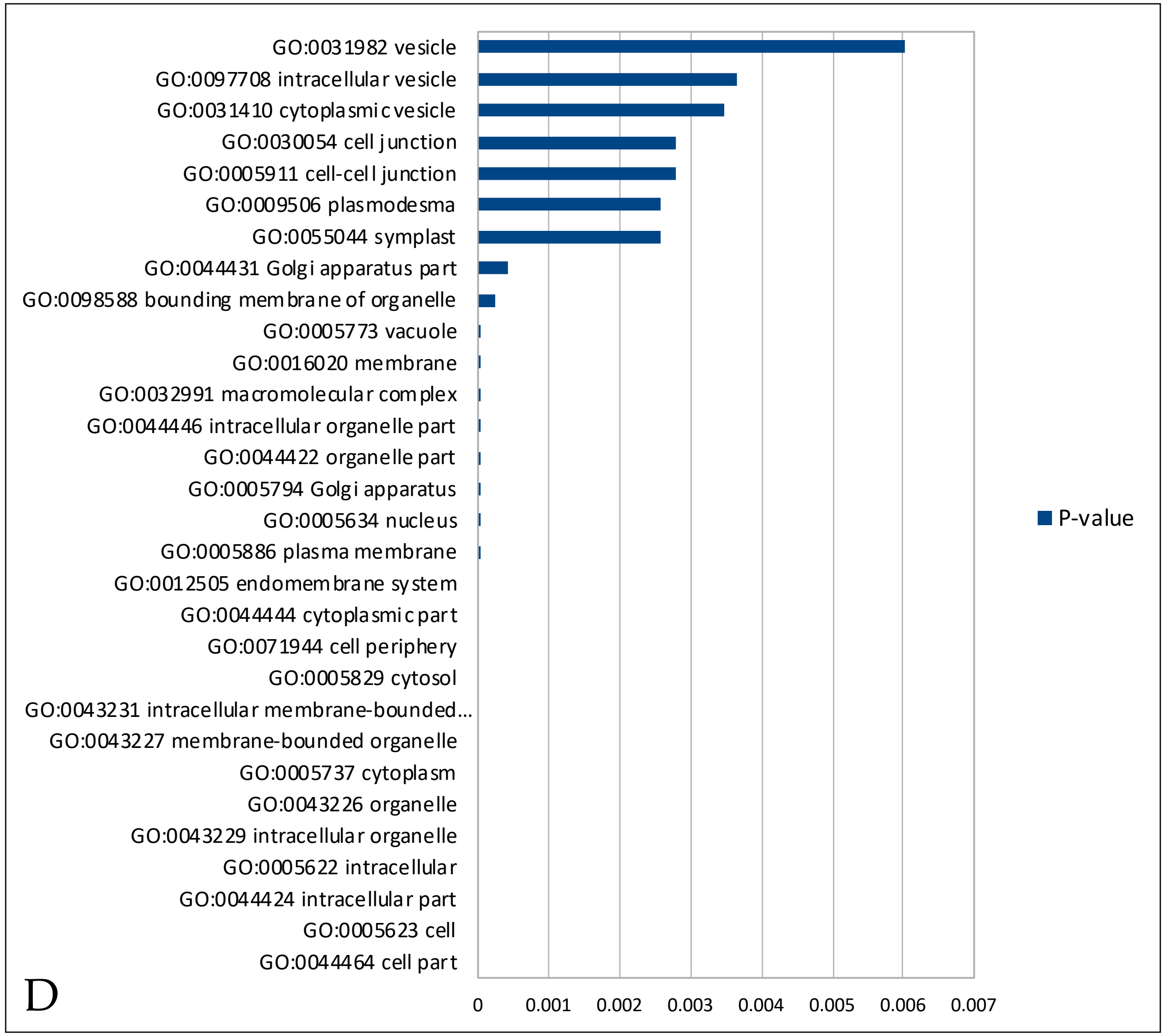

GO:0031982 vesicle
GO:0097708 intracellular vesicle
GO:0031410 cytoplasmic vesicle
GO:0030054 cell junction
GO:0005911 cell-cell junction
GO:0009506 plasmodesma
GO:0055044 symplast
GO:0044431 Golgi apparatus part
GO:0098588 bounding membrane of organelle
GO:0005773 vacuole
GO:0016020 membrane
GO:0032991 macromolecular complex
GO:0044446 intracellular organelle part
GO:0044422 organelle part
GO:0005794 Golgi apparatus
GO:0005634 nucleus
GO:0005886 plasma membrane
GO:0012505 endomembrane system
GO:0044444 cytoplasmic part
GO:0071944 cell periphery
GO:0005829 cytosol
GO:0043231 intracellular membrane-bounded...
GO:0043227 membrane-bounded organelle
GO:0005737 cytoplasm
GO:0043226 organelle
GO:0043229 intracellular organelle
GO:0005622 intracellular
GO:0044424 intracellular part
GO:0005623 cell
GO:0044464 cell part
0
0.001
0.002
0.003
0.004
0.005
0.006
0.007
P-value
D

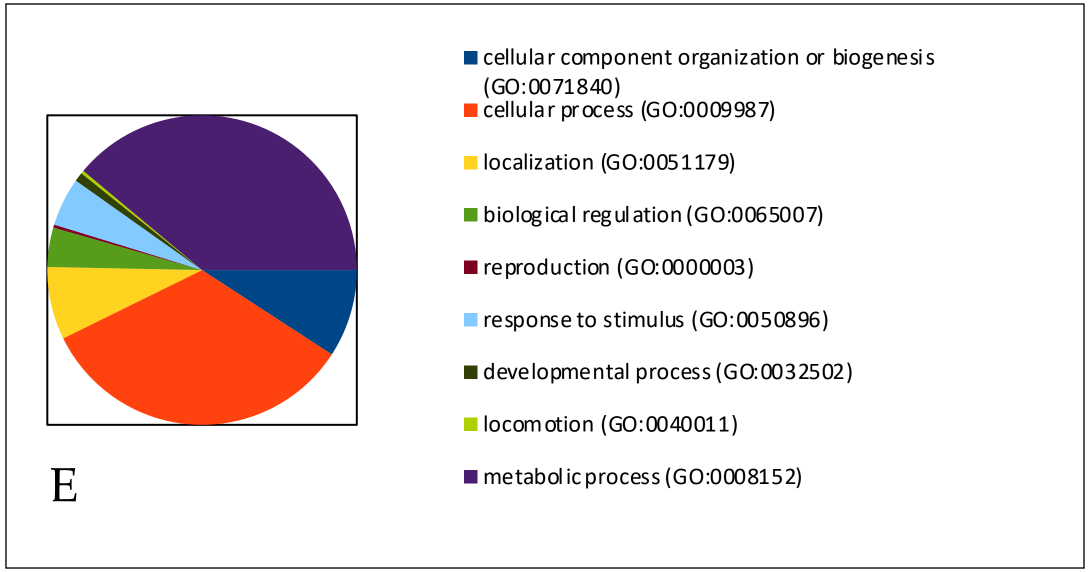

cellular component organization or biogenesis (GO:0071840)
cellular process (GO:0009987)
localization (GO:0051179)
biological regulation (GO:0065007)
reproduction (GO:0000003)
response to stimulus (GO:0050896)
developmental process (GO:0032502)
locomotion (GO:0040011)
metabolic process (GO:0008152)
E

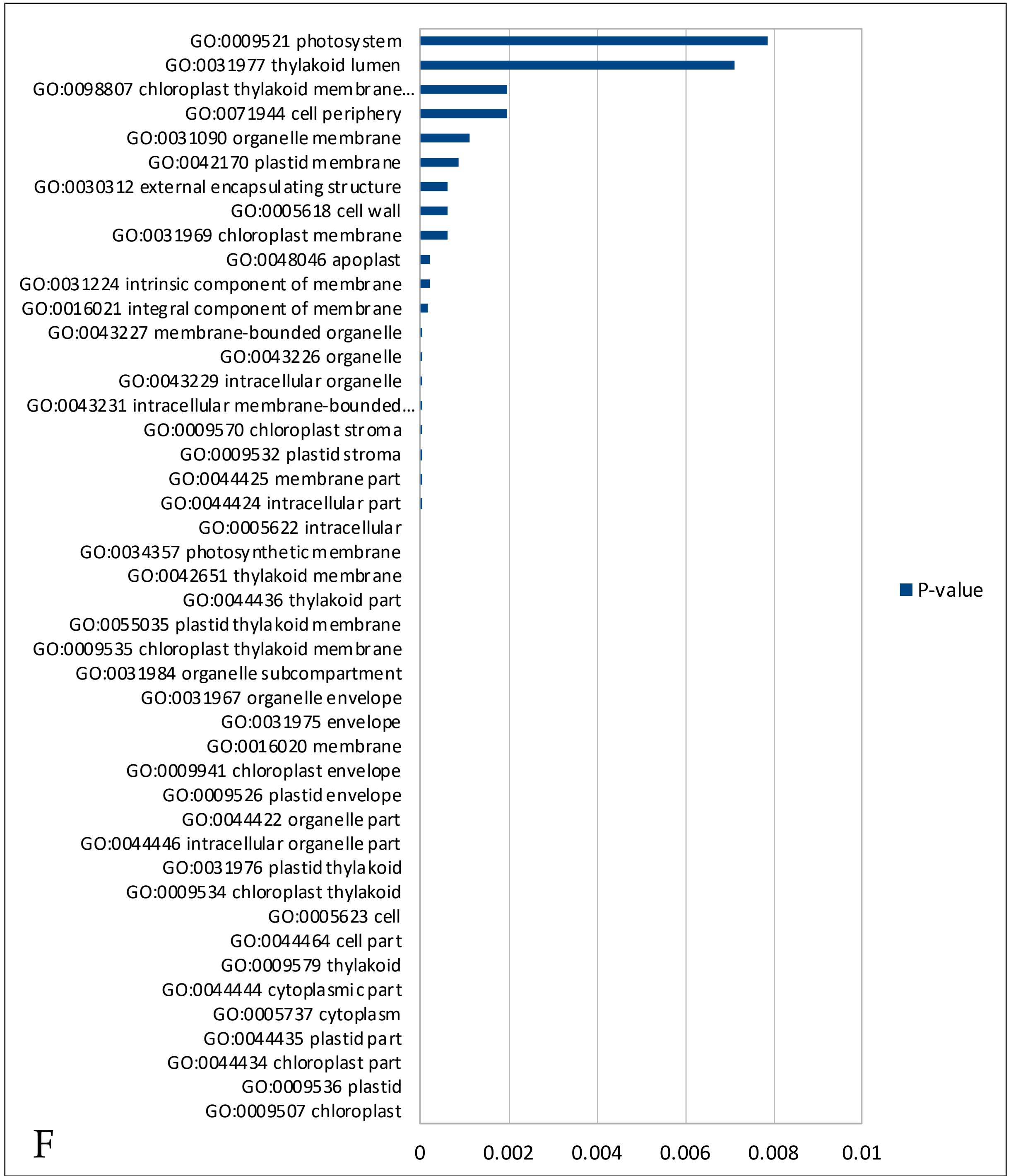
GO:0009521 photosystem
GO:0031977 thylakoid lumen
GO:0098807 chloroplast thylakoid membrane...
GO:0071944 cell periphery
GO:0031090 organelle membrane
GO:0042170 plastid membrane
GO:0030312 external encapsulating structure
GO:0005618 cell wall
GO:0031969 chloroplast membrane
GO:0048046 apoplast
GO:0031224 intrinsic component of membrane
GO:0016021 integral component of membrane
GO:0043227 membrane-bounded organelle
GO:0043226 organelle
GO:0043229 intracellular organelle
GO:0043231 intracellular membrane-bounded...
GO:0009570 chloroplast stroma
GO:0009532 plastid stroma
GO:0044425 membrane part
GO:0044424 intracellular part
GO:0005622 intracellular
GO:0034357 photosynthetic membrane
GO:0042651 thylakoid membrane
GO:0044436 thylakoid part
GO:0055035 plastid thylakoid membrane
GO:0009535 chloroplast thylakoid membrane
GO:0031984 organelle subcompartment
GO:0031967 organelle envelope
GO:0031975 envelope
GO:0016020 membrane
GO:0009941 chloroplast envelope
GO:0009526 plastid envelope
GO:0044422 organelle part
GO:0044446 intracellular organelle part
GO:0031976 plastid thylakoid
GO:0009534 chloroplast thylakoid
GO:0005623 cell
GO:0044464 cell part
GO:0009579 thylakoid
GO:0044444 cytoplasmic part
GO:0005737 cytoplasm
GO:0044435 plastid part
GO:0044434 chloroplast part
GO:0009536 plastid
GO:0009507 chloroplast
0
0.002
0.004
0.006
0.008
0.01
P-value
F

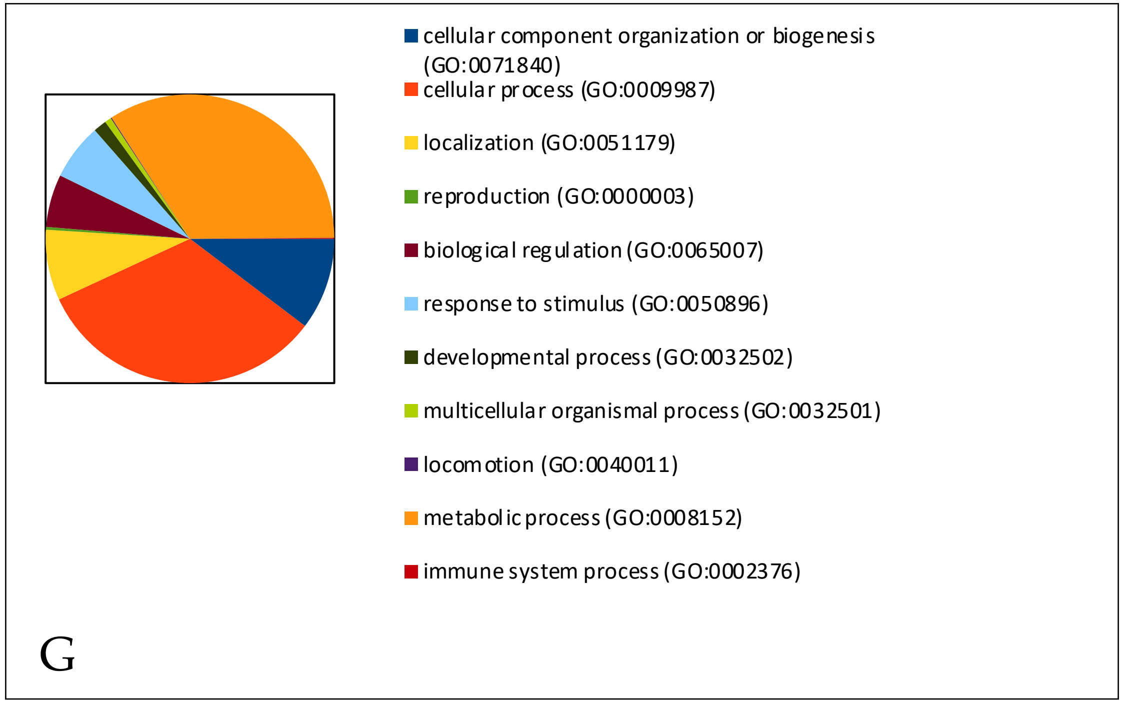
cellular component organization or biogenesis (GO:0071840)
cellular process (GO:0009987)
localization (GO:0051179)
reproduction (GO:0000003)
biological regulation (GO:0065007)
response to stimulus (GO:0050896)
developmental process (GO:0032502)
multicellular organismal process (GO:0032501)
locomotion (GO:0040011)
metabolic process (GO:0008152)
immune system process (GO:0002376)
G

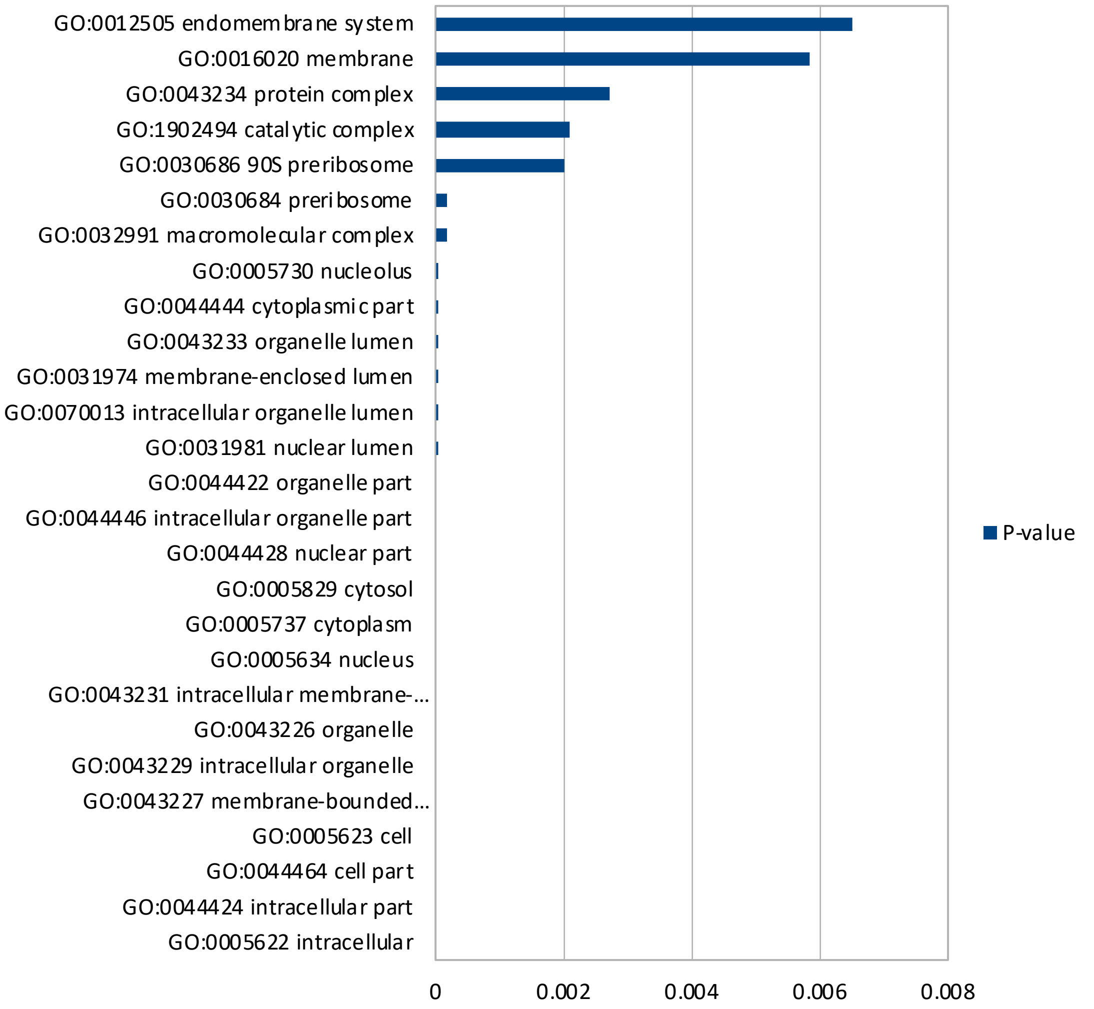
GO:0012505 endomembrane system
GO:0016020 membrane
GO:0043234 protein complex
GO:1902494 catalytic complex
GO:0030686 90S preribosome
GO:0030684 preribosome
GO:0032991 macromolecular complex
GO:0005730 nucleolus
GO:0044444 cytoplasmic part
GO:0043233 organelle lumen
GO:0031974 membrane-enclosed lumen
GO:0070013 intracellular organelle lumen
GO:0031981 nuclear lumen
GO:0044422 organelle part
GO:0044446 intracellular organelle part
GO:0044428 nuclear part
GO:0005829 cytosol
GO:0005737 cytoplasm
GO:0005634 nucleus
GO:0043231 intracellular membrane-...
GO:0043226 organelle
GO:0043229 intracellular organelle
GO:0043227 membrane-bounded...
GO:0005623 cell
GO:0044464 cell part
GO:0044424 intracellular part
GO:0005622 intracellular
0
0.002
0.004
0.006
0.008
P-value

H

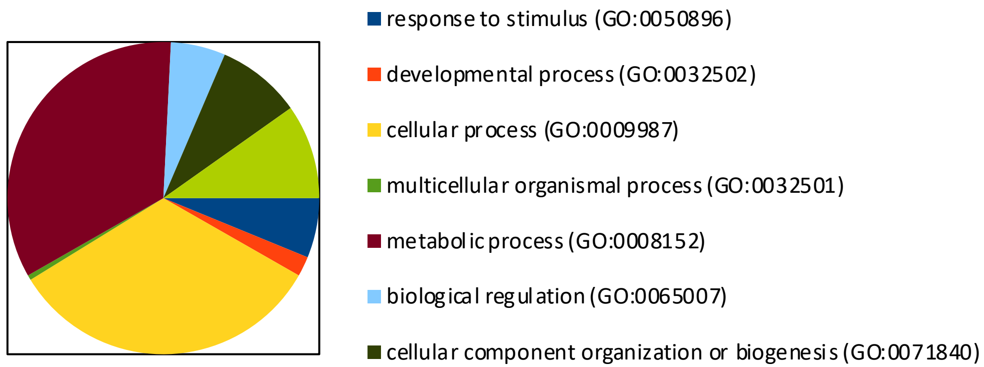
response to stimulus (GO:0050896)
developmental process (GO:0032502)
cellular process (GO:0009987)
multicellular organismal process (GO:0032501)
metabolic process (GO:0008152)
biological regulation (GO:0065007)
cellular component organization or biogenesis (GO:0071840)
localization (GO:0051179)

I

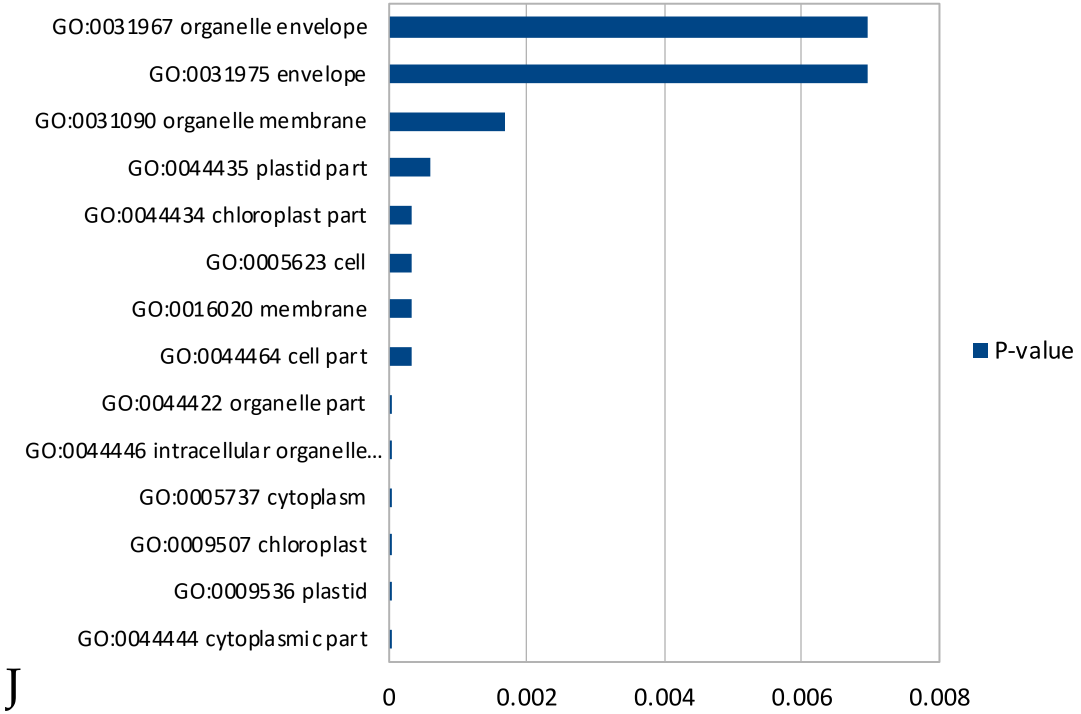
GO:0031967 organelle envelope
GO:0031975 envelope
GO:0031090 organelle membrane
GO:0044435 plastid part
GO:0044434 chloroplast part
GO:0005623 cell
GO:0016020 membrane
GO:0044464 cell part
GO:0044422 organelle part
GO:0044446 intracellular organelle...
GO:0005737 cytoplasm
GO:0009507 chloroplast
GO:0009536 plastid
GO:0044444 cytoplasmic part
0
0.002
0.004
0.006
0.008
P-value

J

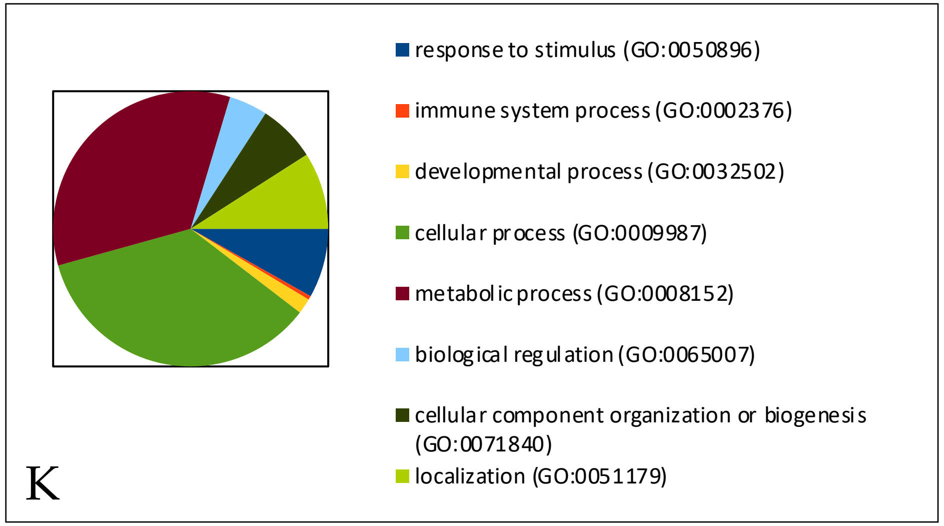
response to stimulus (GO:0050896)
immune system process (GO:0002376)
developmental process (GO:0032502)
cellular process (GO:0009987)
metabolic process (GO:0008152)
biological regulation (GO:0065007)
cellular component organization or biogenesis (GO:0071840)
localization (GO:0051179)
K

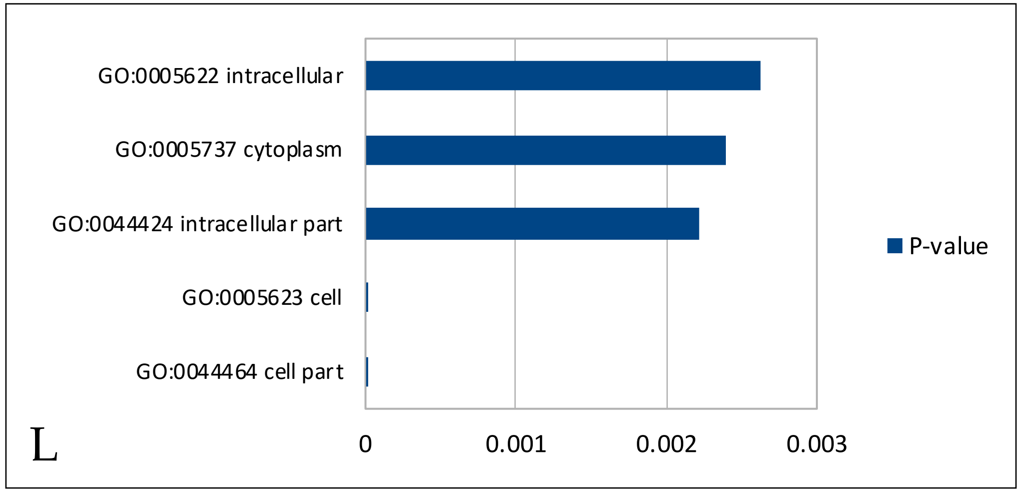
GO:0005622 intracellular
GO:0005737 cytoplasm
GO:0044424 intracellular part
GO:0005623 cell
GO:0044464 cell part
P-value
0
0.001
0.002
0.003
L

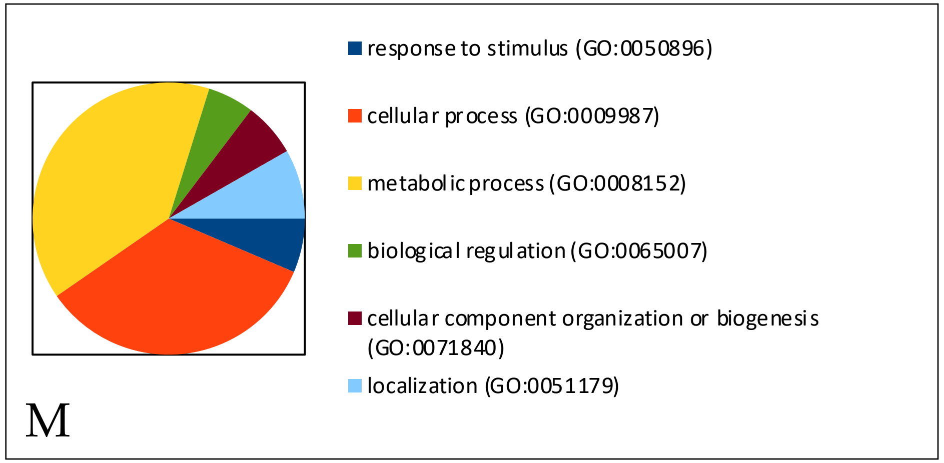
response to stimulus (GO:0050896)
cellular process (GO:0009987)
metabolic process (GO:0008152)
biological regulation (GO:0065007)
cellular component organization or biogenesis (GO:0071840)
localization (GO:0051179)
M

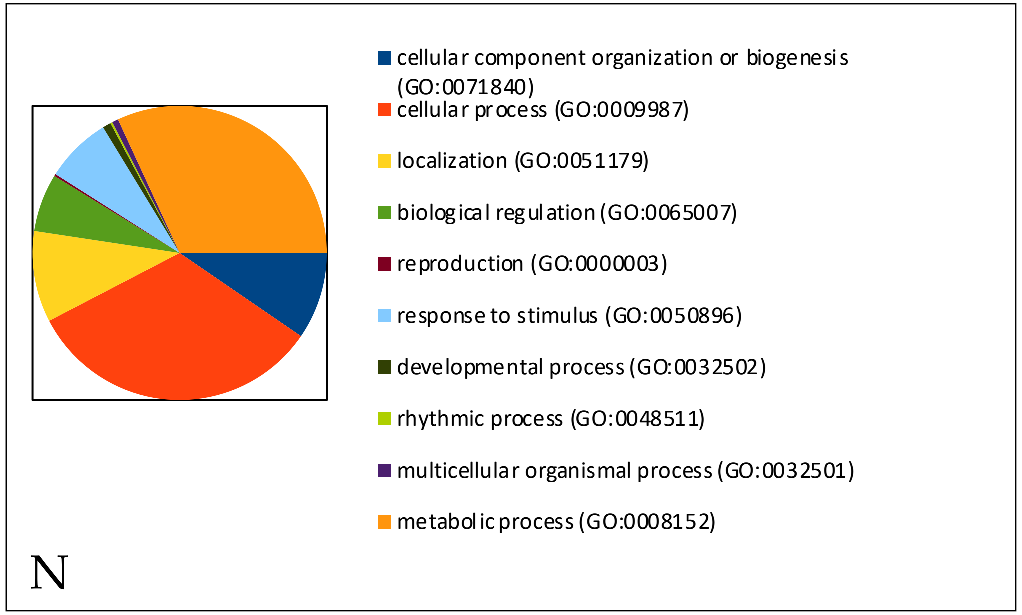
cellular component organization or biogenesis (GO:0071840)
cellular process (GO:0009987)
localization (GO:0051179)
biological regulation (GO:0065007)
reproduction (GO:0000003)
response to stimulus (GO:0050896)
developmental process (GO:0032502)
rhythmic process (GO:0048511)
multicellular organismal process (GO:0032501)
metabolic process (GO:0008152)
N

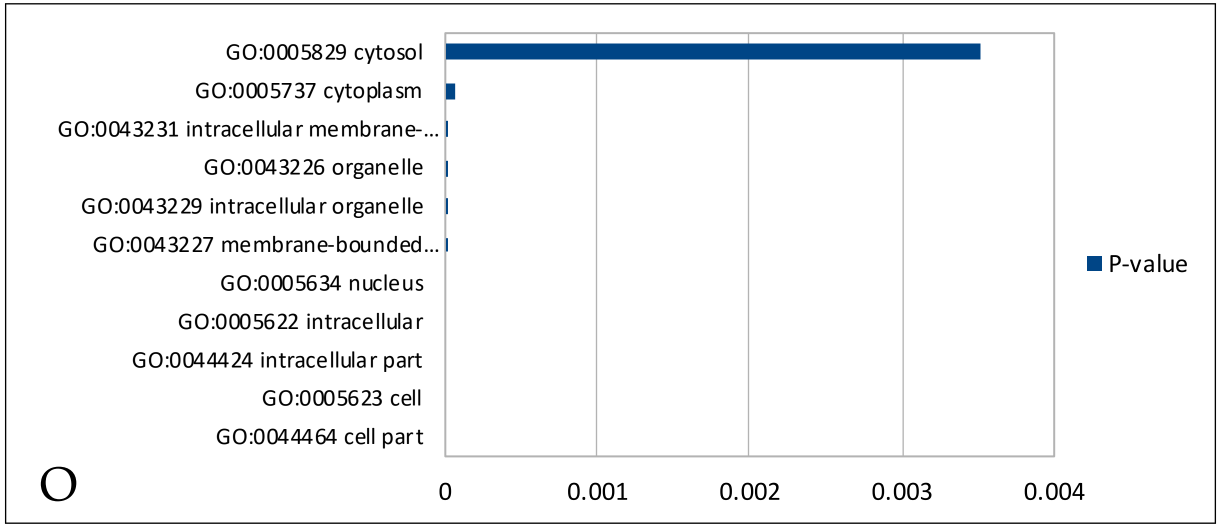

Figure S4. Enrichment of GO terms related to "Biological Processes (BP)" (pie chart) and "Cellular Component (CC)" (horizontal histogram, classes are sorted by *p*-value, most significant at the bottom). **(A)** BP and **(B)** CC of the LR Ch up-expressed subset; **(C)** BP and **(D)** CC of the LR Te up-expressed subset; **(E)** BP and **(F)** CC of the TCR down-expressed subset; **(G)** BP and **(H)** CC of the TCR up-expressed subset; **(I)** BP and **(J)** CC of the TLSR **a** subset; **(K)** BP and (**L**) CC of the TLSR **b** subset; **(M)** BP the TLSR **c** subset; and (**N**) BP and (**O**) CC of the TLSR **d** subset.

Table S1. Statistical overview of sequencing and transcriptome assembly data.

Items	Trinity	TransRate
Number of libraries	24	24
Total number of clean reads	793,583,651	743,950,152
Number of reads in Champagne	432,811,373	405,452,636
Number of reads in Terese	360,772,278	338,497,516
# contigs present only in Champagne	5,845	5,689
# contigs present only in Terese	6,257	5,186
# contigs (>= 0 bp)	150,342	122,194
# contigs (>= 1000 bp)	67,384	49,454
Total length (>= 0 bp)	176,909,219	133,129,560
Total length (>= 1000 bp)	137,299,244	99,332,177
# contigs	100,020	76,267
Minimum length of contigs (bp)	201	201
Average length of contigs (bp)	1,177	1,089
Largest contig (bp)	12,528	11,608
Total length	161,140,190	118,787,279
GC (%)	38.13	38.04
N50	1,961	1,904
N75	1,294	1,242
L50	27,830	21,098
L75	52,994	40,289
# N's per 100 kbp	0	0
Number of unigenes	81,321	73,225

Table S2. KEGG pathways repartition in each subset of DEGs, divided into two sub-categories of KEGG classification. The Ch up and Te up correspond to "Line Response" followed by two columns of "Temperature Common Response" and the last four columns referring to the "Temperature Line Specific Response".

	**Ch up**	**Te up**	**TCR down**	**TCR up**	**TLSR a**	**TLSR b**	**TLSR c**	**TLSR d**
1. Metabolism								
1.0 Global and overview maps								
Metabolic pathways	114	106	82	55	38	28	10	27
Biosynthesis of secondary metabolites	75	66	57	31	30	17	7	13
Carbon metabolism	11	18	16	7	5	3	3	1
2-Oxocarboxylic acid metabolism	2	3	4	5	2	2		1
Fatty acid metabolism	3	1	4	2	3	3		
Biosynthesis of amino acids	16	14	10	9	4	4	1	2
1.1 Carbohydrate metabolism								
Glycolysis / Gluconeogenesis	6	10	5	3	1	1		1
Citrate cycle (TCA cycle)	2	2	2	5		2		
Pentose phosphate pathway		7	3				2	1
Pentose and glucuronate interconversions	2	4					1	1
Fructose and mannose metabolism	1	8	1		1			2
Galactose metabolism	3	5		2				
Ascorbate and aldarate metabolism	5	3	4		1		1	1
Starch and sucrose metabolism	8	10	5	8	6	3	1	2
Amino sugar and nucleotide sugar metabolism	8	9	5	2	4		1	
Pyruvate metabolism	7	9	3	2	2	1	1	1
Glyoxylate and dicarboxylate metabolism	3	4	10	1	4	2		
Propanoate metabolism			2	1	2		2	
Butanoate metabolism	1		1	1	1	1		
C5-Branched dibasic acid metabolism		1		2				
Inositol phosphate metabolism	2	5	1	2	1	4		
1.2 Energy metabolism								

Oxidative phosphorylation	6	3	4	1	6	1	1	
Photosynthesis	2	1	7					
Photosynthesis - antenna proteins	1	2	1	2			1	
Carbon fixation in photosynthetic organisms	4	4	4		1		1	1
Nitrogen metabolism	4	1	1		1			1
Sulfur metabolism	3	2	2		1			
1.3 Lipid metabolism								
Fatty acid biosynthesis	2		1	1	1	2		
Fatty acid elongation	3	1	4		3		1	
Fatty acid degradation	4	3		2	2	3		
Synthesis and degradation of ketone bodies			1		1			
Cutin, suberine and wax biosynthesis	1	2			2	1	1	
Steroid biosynthesis	2	2	1		1			
Glycerolipid metabolism	8	6	3	4		2	1	2
Glycerophospholipid metabolism	8	7	4	5	2	4	1	5
Ether lipid metabolism		2		2	1	1		1
Sphingolipid metabolism	2	1	1	4		1		1
Arachidonic acid metabolism	2			1		1		
Linoleic acid metabolism	1			1				1
alpha-Linolenic acid metabolism	4	3	3	1		1		1
Biosynthesis of unsaturated fatty acids	1	2	3	1	1	1		
1.4 Nucleotide metabolism								
Purine metabolism	8	11	3	9	3	5		3
Pyrimidine metabolism	5	4		5	1	4		6
1.5 Amino acid metabolism								
Alanine, aspartate and glutamate metabolism	6	1	2	2	1	1		2
Glycine, serine and threonine metabolism	5	4	6	2	2			1
Cysteine and methionine metabolism	13	3	2	1	2	1		1
Valine, leucine and isoleucine degradation	1	5	3	1	1		2	1
Valine, leucine and isoleucine biosynthesis		2		2				
Lysine biosynthesis	2	1						1

Lysine degradation	1	5		1	1			
Arginine biosynthesis	4	1	4		1			1
Arginine and proline metabolism	3	3	2	2	1	2		
Histidine metabolism	1	1		2				1
Tyrosine metabolism	5	3		1	3	1		1
Phenylalanine metabolism	4	2	3	1	2	1		2
Tryptophan metabolism	3	5	3	1	2	1		
Phenylalanine, tyrosine and tryptophan biosynthesis	5	1	2	1	2	1		1
1.6 Metabolism of other amino acids								
beta-Alanine metabolism	4	2		1	1	2	1	
Taurine and hypotaurine metabolism	1	1				1		
Phosphonate and phosphinate metabolism	1			2				1
Selenocompound metabolism	3							1
Cyanoamino acid metabolism	3	5	4	3	3	3		1
Glutathione metabolism	9	2	2	4	1	2	1	2
1.7 Glycan biosynthesis and metabolism								
N – Glycan biosynthesis	2	3			1			2
Other types of O – glycan biosynthesis							1	
Glycosaminoglycan degradation		1	1					
Glycosylphosphatidylinositol (GPI)-anchor biosynthesis	1	2		1				1
Glycosphingolipid biosynthesis - globo and isoglobo series	1	1	1					
Glycosphingolipid biosynthesis - ganglio series			1					
Other glycan degradation	2		1	2			1	1
1.8 Metabolism of cofactors and vitamins								
Thiamine metabolism	1		1	1		1		
Riboflavin metabolism	2	1						
Vitamin B6 metabolism	1		1	1				
Nicotinate and nicotinamide metabolism	2	1		1	1			2
Pantothenate and CoA biosynthesis	1			2				1

Biotin metabolism	1							
Lipoic acid metabolism	1							
Folate biosynthesis		1	2				1	1
One carbon pool by folate	1	1	5		1			
Porphyrin and chlorophyll metabolism	3	1	3	1	4	1		
Ubiquinone and other terpenoid-quinone biosynthesis		1	4			2		1
1.9 Metabolism of terpenoids and polyketides								
Terpenoid backbone biosynthesis	4	2	4	1	2		1	2
Monoterpenoid biosynthesis	1	1						
Sesquiterpenoid and triterpenoid biosynthesis	1	2						
Diterpenoid biosynthesis		2	1					
Carotenoid biosynthesis	2		4		1			1
Brassinosteroid biosynthesis		1		1				
Zeatin biosynthesis	1	1	1		1			
1.10 Biosynthesis of other secondary metabolites								
Phenylpropanoid biosynthesis	9	12	9	5	4	4		2
Stilbenoid, diarylheptanoid and gingerol biosynthesis			1					
Flavonoid biosynthesis	5		2		1			
Flavone and flavonol biosynthesis	2				1			
Anthocyanin biosynthesis					1			
Indole alkaloid biosynthesis	2							
Isoquinoline alkaloid biosynthesis	3	1			2			
Tropane, piperidine and pyridine alkaloid biosynthesis	3	1		1	3			1
Betalain biosynthesis	1							
Glucosinolate biosynthesis			1	1	1			
Monobactam biosynthesis								1
Sub-toatal	467	425	334	222	179	122	45	108
2. Genetic Information Processing								
2.1 Transcription								

RNA polymerase	1	2		4		3		
Basal transcription factors	1	4	1	1				1
Spliceosome	5	11	3	18	2	2		5
2.2 Translation								
Ribosome	6	25		2		1		4
Aminoacyl-tRNA biosynthesis	3	2		2				2
RNA transport	5	11	1	13		4		6
mRNA surveillance pathway		8		14		3		7
Ribosome biogenesis in eukaryotes	2	2		21		1		5
2.3 Folding, sorting and degradation								
Protein export	2	3		2	1		1	1
Protein processing in endoplasmic reticulum	5	8	4	9	4	3	1	3
SNARE interactions in vesicular transport	1	5		2				1
Ubiquitin mediated proteolysis	4	13	3	4	1		2	2
Sulfur relay system		1				2		2
Proteasome	2	1		6		3		
RNA degradation	2	6	2	10				1
2.4 Replication and repair								
DNA replication	3	1		1	1	2		1
Base excision repair	2	6	1	1		1		
Nucleotide excision repair	4	4		2	1	2		
Mismatch repair	4	1			1	2		
Homologous recombination	1	5				2		1
Non – homologous end – joining		1		1				1
3. Environmental Information Processing								
3.1 Membrane transport								
ABC transporters	1	3		2		1		1
3.2 Signal transduction								
MAPK signaling pathway - plant	3	8		4			1	3
Phosphatidylinositol signaling system	2	6	1	3		4	1	
Plant hormone signal transduction	4	17	3	7	1	1	2	6

4. Cellular Processes								
4.1 Transport and catabolism								
Endocytosis	5	14	3	12	3	4		6
Phagosome	3	4	4	5	2		2	
Peroxisome	4	5	3	6	1	3	1	1
Autophagy - other	2	3		1				2
5. Organismal Systems								
5.1 Environmental adaptation								
Circadian rhythm - plant	2	2	2	6	3	2		3
Plant-pathogen interaction	7	8		6	1	2	1	
6. Human Diseases								
6.1 Endocrine and metabolic diseases								
AGE–RAGE signaling pathway in diabetic complications		1	1	1				
**Sub-total**	**86**	**191**	**32**	**166**	**22**	**48**	**12**	**65**
**Total**	**553**	**616**	**366**	**388**	**201**	**170**	**57**	**173**
**Total number of genes in each subset**	**906**	**1581**	**520**	**883**	**253**	**228**	**131**	**479**

Table S3. Kinases repartition in each subset of DEGs, using iTAK software. The Ch up and Te up correspond to "Line Response" followed by two columns of "Temperature Common Response" and the last four columns referring to the "Temperature Line Specific Response".

**Kinases**	**Ch up**	**Te up**	**TCR down**	**TCR up**	**TLSR a**	**TLSR b**	**TLSR c**	**TLSR d**
ATN1 Like Family				1				
Calcium Dependent Protein Kinase	1	3		2		1		1
Casein Kinase I Family			1	5				3
CDC2 Like Kinase Family	2	3		2		1		
CRPK1 Like Kinase (Types 1 and 2)	3				1			
CTR1/EDR1 Kinase		3						1
Domain of Unknown Function 26 (DUF26) Kinase	4	2	1		1			
ELM1/PAK1/TOS3 Like Kinase		2						
GmPK6/AtMRK1 Family		4	2	1		1		1
IRE/NPH/PI dependent/S6 Kinase		1		3			1	2
LAMMER Kinase Family			1					
Legume Lectin Domain Kinase		2	1	1		1		
Leucine Rich Repeat Kinase I	1	1						1
Leucine Rich Repeat Kinase II & X		2					1	1
Leucine Rich Repeat Kinase III		2	3		2			
Leucine Rich Repeat Kinase IX	1	3						
Leucine Rich Repeat Kinase VI				1				
Leucine Rich Repeat Kinase X		1						
Leucine Rich Repeat Kinase XI & XII	1	7	2	1	1			3
Leucine Rich Repeat Receptor Kinase		1		1				
Leucine Rich Repeat Receptor VIII		1						
Leucine-rich transmembrane protein kinase			1					
LRK10 Like Kinase (Type 1)	2					1		
MAP2K	1	2	1	2				
MAPK Family	1	2	1	2				
Other Protein Kinase		1						
Plant External Response Like Kinase		4						1
Possible MAP2K		4						
Putative LRR receptor-like protein kinase		1		1				1
Putative receptor like protein kinase	2							
Receptor Like Cytoplasmic Kinase I		1						
Receptor Like Cytoplasmic Kinase II								1
Receptor Like Cytoplasmic Kinase IV		1						1
Receptor Like Cytoplasmic Kinase IX		1						
Receptor Like Cytoplasmic Kinase V		2		2				
Receptor Like Cytoplasmic Kinase VI				1				1
Receptor Like Cytoplasmic Kinase VII	2		1	3	1	1		3
Receptor Like Cytoplasmic Kinase VIII		1						
Receptor-like protein kinase	1	1		1	1	1		
RKF3 Like Kinase		1						
S Domain Kinase (Type 1)		1		1		2		
S Domain Kinase (Type 2)	4					1		
SNF1 Related Protein Kinase (SnRK)		5	1	3		1		1
STE20-PAK Like Protein Kinase				1				
Tousled like kinase								1
Unknown Function Kinase		4		1				
Wall Associated Kinase-like Kinase				2				
**Total**	**26**	**70**	**16**	**38**	**7**	**11**	**2**	**23**
